# Assessment of monitoring systems in the management of severe acute malnutrition in northern Nigeria

**DOI:** 10.1186/s40795-020-00405-z

**Published:** 2021-01-12

**Authors:** Veronica Tuffrey, Cora Mezger, Simeon Nanama, Assaye Bulti, Gloria Olisenekwu, Charles Umar, Emma Jones, Esther Namukasa

**Affiliations:** 1 School of Life Sciences, University of Westminster, 115 New Cavendish Street, London, W1W 6UW UK; 2 Department of Statistics, University of Oxford, 24-29 St Giles, Oxford, OX1 3LB UK; 3 UNICEF, United Nations House, Plot 617/618, Diplomatic Drive, Central Business District, PMB 2851, Garki, Abuja, Nigeria; 4 Oxford Policy Management Nigeria, House 2, No.16 Mafemi Crescent, Utako, Abuja, Nigeria; 5 Oxford Policy Management Limited, Clarendon House, Level 3, 52 Cornmarket Street, Oxford, OX1 3HJ UK

**Keywords:** Assessment, Monitoring, Malnutrition, Data, Data quality, Nigeria, Community-based management of acute malnutrition, CMAM

## Abstract

**Background:**

In northern Nigeria, UNICEF has supported introduction of a short message service (SMS) system for data transmission in the Community-based Management of Acute Malnutrition (CMAM) programme. The SMS system operates in parallel to the traditional paper-based system, and weekly data are transmitted directly from the health facilities to the federal level. For the paper system, monthly data summaries are passed through all levels of government. We assessed the data quality and performance of both CMAM information systems.

**Methods:**

We undertook a contextualised study in one state in north-west Nigeria, with additional analysis of secondary data from five states. Fieldwork methods included: observation of the data system in nine selected facilities in three local government areas; recounting of data for admissions, exits, and ready-to-use therapeutic food (RUTF) utilisation; and interviews with health workers and government officials.

**Results:**

While the small number of facilities does not enable robust generalisation of the quantitative findings, the strengths and weaknesses detected pertain to the whole programme, as they relate to how the system was designed and is operated. We found that the accuracy and reliability of CMAM data were deficient to a similar extent in the paper-based and SMS systems. For the audited month, we found large discrepancies between recounted data and paper records in regard to admissions, exits and RUTF cartons consumed in the majority of facilities visited. There was also a large discrepancy in the reported percentage of “deaths or defaulters” (6.5%) compared to 22% based on a recount of outpatient cards. Errors are mainly introduced during data collection and when completing tallies at facility level.

**Conclusion:**

Our findings indicate the need for improvements in the design of the monitoring system, training and supervision of data management, and communication of results; as well as clear evidence on how measures to improve data quality may affect performance of individual CMAM clinics. The CMAM default and death rates currently reported in Nigeria are likely to be under-estimates, and therefore provide a misleadingly good impression of CMAM programme performance.

**Supplementary Information:**

The online version contains supplementary material available at 10.1186/s40795-020-00405-z.

## Background

Each year in Nigeria, more than two and a half million children under age five are affected by severe acute malnutrition (SAM), or extremely low weight-for-height (wasting), a condition that if untreated would lead to nearly half a million deaths [1]. There is large geographical variation in malnutrition rates in Nigeria. Rates of global acute malnutrition (GAM) are highest in the North-East (8.7%) and North-West zones (8.3%) [2]. The nutrition situation is particularly serious in the northern states of Jigawa, Katsina, Kebbi, Sokoto, Yobe and Zamfara, where more than half of under 5-year-old children are stunted (low height-for-age) [2].

Programmatic approaches to treatment of severe acute malnutrition (SAM) in children are centred on provision of nutrition-dense foods to promote weight gain, alongside parental counselling on infant care and feeding. Over the past two decades, the advent of ready to use therapeutic foods (RUTF) has enabled a shift from in-patient to out-patient treatment of SAM [3]. This approach is termed Community-based Management of Acute Malnutrition (CMAM). In this model, community health workers or volunteers actively find cases of wasting within the community, and international guidelines state that children should be followed up periodically after discharge from treatment to avoid relapse [4].

CMAM was introduced in northern Nigeria in 2009, supported by development partners such as UNICEF, Save the Children, Médecins Sans Frontières and Action Against Hunger. These partners work closely with the federal and state governments to provide CMAM services within the public health system in targeted facilities. Funding from the Children’s Investment Fund Foundation (CIFF), the UK Department for International Development and the European Union have more recently supported efforts to scale up CMAM services [5]. Now the CMAM programme is provided in twelve of the thirteen north-eastern and north-western states. In 2014, a coverage survey estimated that 37% of children eligible for care received treatment [6].

In Nigeria, malnourished children admitted to the CMAM programme are treated weekly at outpatient clinics, with only the most severe cases admitted for inpatient care. At each visit, the child’s nutrition status is monitored, the carer is counselled, and the child is provided with essential drugs, vitamin A supplementation, vaccination (if required) and sufficient RUTF to last until the following visit [3]. Mid-upper arm circumference (MUAC) is used as the main criterion for admission and discharge of children to the programme [7].

Until 2016, the Nigeria CMAM programme used a paper-based system for data management, to support programme planning and monitoring. Since 2016, UNICEF supported the government of Nigeria to introduce an additional smartphone-based system with the aim to improve the standardisation, transfer and speed of reporting of monitoring information using Short Messages Service (SMS), based on the open source RapidPro platform [8]. At the time of the study, the SMS system included 827 facilities out of the 1511 that offer the CMAM programme in the country.

**Fig. 1:**
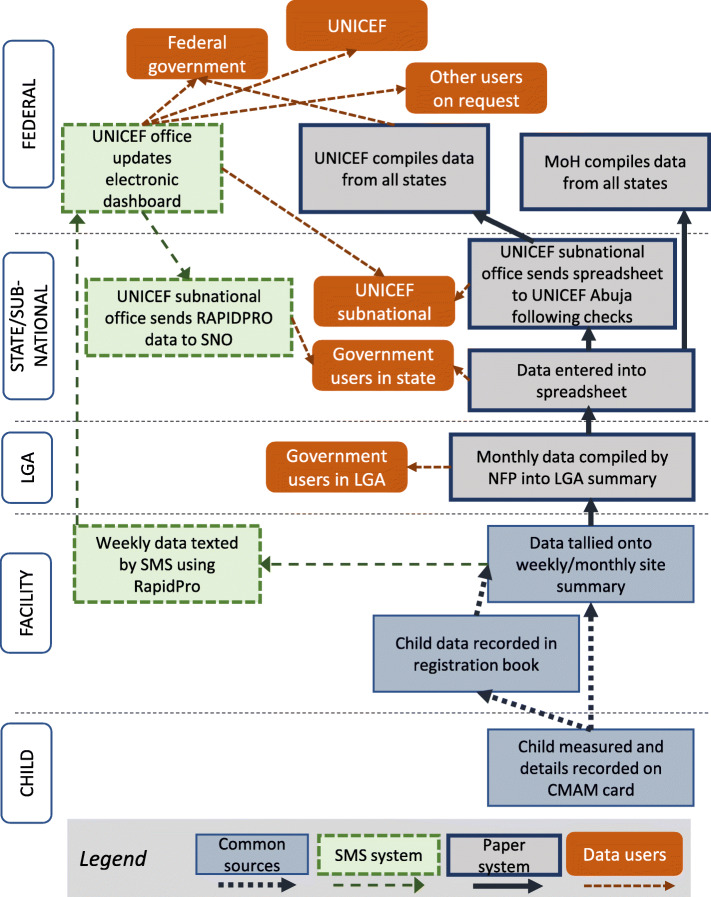
CMAM data flow. Abbreviations: CMAM: Community-based management of acute malnutrition; LGA: Local Government Area; NFP: Nutrition Focal Person; SMS: Short message service; SNO: State Nutrition Officer

Figure 1 illustrates the data flows for the paper-based and SMS systems. In the paper-based system, on a monthly basis, paper forms with aggregated data are transferred from health facilities to the Nutrition Focal Person (NFP) at local government authority (LGA) level. The NFP, in turn, transfers the data to the State Nutrition Officer (SNO). At state level, the data are entered into a spreadsheet, and in this form, data are transferred to federal level via UNICEF. With the new SMS system, weekly aggregated routine data are also texted from facility level directly up to UNICEF at the federal level.

With respect to the information flow back down between levels, in addition to transfer of the RapidPro datafile from federal to state level (as shown on Fig. 1), there is some degree of feedback to the LGA and facility levels at monthly state-level meetings, where data quality and service performance are discussed. At state and federal levels, the CMAM data are to some extent used for monitoring, planning, reporting, tracking, and advocacy. At LGA and facility levels, CMAM data are used predominantly for forecasting, to ensure timely replenishment of RUTF supplies.

In 2017, Oxford Policy Management (OPM) undertook an assessment of the quality and use of CMAM routine monitoring data and of the performance of the underlying paper-based and SMS systems. The objective was to support improvements to the CMAM programme. The assessment was undertaken in the North-West of the country, in collaboration with UNICEF and with funding support from CIFF. Earlier coverage surveys undertaken in 2013/2014 in various states have highlighted problems with the quality of the CMAM programme data [9–16]. For example, some surveys indicated that MUAC measurements were often poorly carried out [9, 10], protocols for recovery stated in the national CMAM guidelines [17] were not being consistently applied [11, 12], and that outcome indicators (recovery, defaulter, death) and some admissions data were inaccurate [13–16]. Discrepancies were found between data extracted from facility level records and data collated at LGA level [13, 15]. Data related to RUTF were frequently found to be missing from reported data [18]. RUTF misappropriation has been noted to be a problem in some areas of northern Nigeria, which may promote the falsification of monitoring data and has prompted some state governments to establish ‘RUTF Task Forces’ and community oversight committees for the CMAM programme [19].

Data of good quality are needed to inform decision-making during programme monitoring, review, planning, and improvement; advocacy; and policy development and review [20]. This study aimed to enhance, complement and update previous findings with respect to data quality in the CMAM programme in northern Nigeria by using a tailored methodology to assess the entire CMAM data collection, transmission and analysis process.

## Methods

**Fig. 2:**
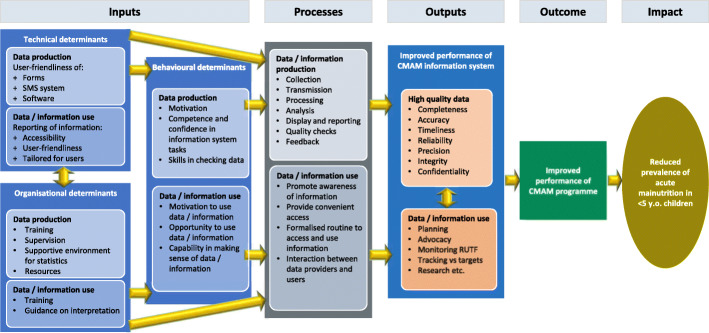
Conceptual framework for assessment of CMAM information system, Nigeria, adapted from the PRISM framework

Figure 2 shows the conceptual framework used in the study. It is based on the PRISM (Performance of Routine Information System Management) framework developed by the Measure Evaluation Project [21]. This framework was appropriate because it includes the institutional environment of information systems, not just their products and key data production processes. PRISM assesses performance in terms of data quality and information use separately, and identifies underlying technical, organizational, and behavioural reasons for strengths and weaknesses in these two elements. We amended the PRISM framework to be pertinent for CMAM in Nigeria; and incorporated insights on the use of evidence in decision-making, which depends on intermediary behavioural factors such as capability, motivation and opportunity [22]. In this paper, we discuss the findings related to the accuracy, reliability, completeness and timeliness of data.

We selected nine health facilities in three LGAs, in one northern Nigerian state. The number of facilities included was constrained by budgetary considerations. Nine facilities provided a range of size, performance, and peri-urban versus rural location, as shown in Table 1. Their selection was also influenced by logistic constraints including security and the day of the week on which the CMAM clinic was held.

**Table 1:** Characteristics of the selected facilities

Facility	Average number of children in treatment at beginning of month (2016)	Average number of monthly admissions (children 6-59 m) (2016)	% recovered/ discharged^a^ (2016)	Location	Approx. distance from state capital in km
0 (pre-test)	167	65	96%	Urban	< 20
1	74	42	94%	Urban	< 20
2	100	60	89%	Urban	< 20
3	90	47	94%	Urban	< 20
4	120	41	98%	Rural	20–80
5	66	23	97%	Rural	> 80
6	104	39	96%	Rural	20–80
7	141	67	94%	Rural	20–80
8	125	53	74%	Rural	> 80
9	97	49	93%	Rural	> 80
**All CMAM in state**	**117**	**50**	**93%**		

Notes ^a^ “discharged” includes: recovered, deaths, defaults, non-recovered

We adopted a mixed-methods approach for primary data collection (both qualitative and quantitative) because a key goal was to identify potential means for system improvement. A large-scale solely quantitative study may have better revealed the extent and distribution of data quality problems, but a shallower understanding of their origins. We used a combination of tools and analytical approaches to explore the components of data quality depicted in the conceptual framework (inputs, processes and outputs) for both the paper and SMS-based data systems. This paper describes the findings from the quantitative verification. Data use and constraints to usage were also assessed but are not covered in this article.

The data sources and study methods included:

- Desk-based review of data collection and transmission forms and documentation (guidelines, training material and protocols);
- Primary data collection (November–December 2017) in nine facilities.
  - Observation of the inputs and processes relating to the monitoring system in health facilities and semi-structured key-informant interviews with health workers and government officials at facility, LGA, state and federal level; observations and interviews were collated and synthesised according to the items in the conceptual framework. The interview guide (Additional_file_1) was developed for the study in part by adapting PRISM tools developed by the Measure Evaluation Project [21].
  - Recount of data from the OTP (Outpatient Therapeutic Programme) cards for admissions, exits, and records for RUTF utilisation at these facilities for a specified time window in 2017;
- Descriptive analysis of facility-level data from the paper-based and the SMS system provided by UNICEF for the period of January 2017 to July 2017 from five states, to examine completeness, aspects of reliability and accuracy in terms of internal consistency checks, outliers and trends to examine consistency between performance variables, as well as timeliness.
- Descriptive analysis to compare facility-level counts of admissions, exits and RUTF extracted from the paper-based and SMS-based monitoring systems against data recounted from source forms in the subset of facilities visited during fieldwork.

Fuller details on methods are available from an alternative source [23]. This paper summarises the findings relating to the quality dimensions for which quantitative data were collected (completeness, accuracy, timeliness, reliability) and draws on findings from observations and interviews to suggest reasons for patterns identified. For more detailed findings relating to these four dimensions, findings relating to those quality dimensions that are not quantifiable (integrity, confidentiality, relevance and accessibility), and findings relating to use of the CMAM data, readers are referred to alternative sources [24, 25].

## Results

### Data accuracy and reliability: findings from the data verification

We compared recounts of data from OTP cards performed during our visits to facilities, with data from the paper records held by the facilities for July 2017.

#### Admissions data

**Fig. 3:**
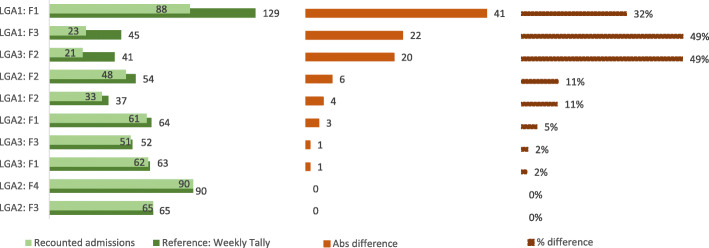
Findings from verification of admissions data for one month from 10 CMAM facilities framework

Figure 3 illustrates the findings from the data verification relating to admissions. It compares the numbers recorded on paper in the weekly tallies with the number of admissions we recounted from the OTP cards for the month of July 2017. Discrepancies were greater than 10% for half of the selected ten facilities, and greater than 30% in three facilities, with a maximum value of 49%.

#### CMAM exit data

**Fig. 4:**
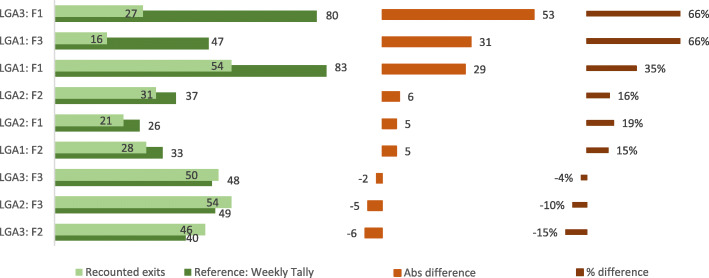
Findings from verification of discharge data for one month from 9 CMAM facilities

Figure 4 shows findings from data verification relating to exits from treatment, where exits are defined as in the CMAM guidelines [17] as cured, defaulter, death, non-recovered (did not meet the discharge cured criteria after 4 months in treatment) or transferred to inpatient care or another OTP. The figure compares numbers recorded in the weekly tallies with the number of exits recounted from the OTP cards for July 2017. Discrepancies were greater than 10% for eight of the nine visited facilities, and greater than 30% in three facilities, with a maximum value of 66%.

We also intended to recount exits by the same exit categories used on the tally forms (recovered, death, defaulter, non-recovered or transferred). However, this task was not straightforward due to gaps in data recorded in the “outcome” cell on the OTP cards. Hence where outcome data were missing, we used relevant data from elsewhere in the card to identify the most likely exit category. We combined defaulters and deaths, because without a functioning system of follow-up after discharge it is impossible to know if a defaulted beneficiary is alive and thus a ‘true’ defaulter, or if they have died. The verification suggested a considerably higher number of defaulters or deaths than were reported in the electronic databases. For July 2017, the reported percentage of “deaths or defaulters” was 29 children or 6.5% of all exits reported through the CMAM system, compared to 72 children or 22% based on the recount of OTP cards.

#### RUTF consumption

**Fig. 5:**
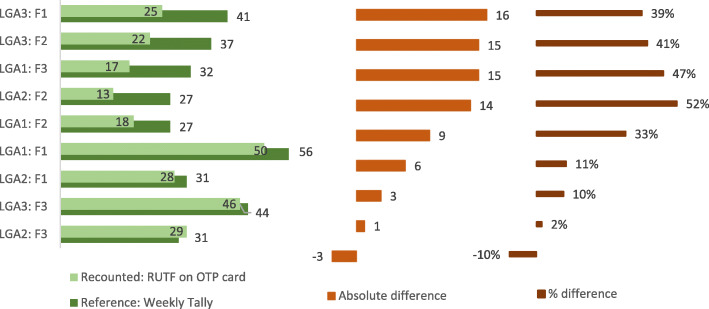
Findings from verification of RUTF data for one month from 9 CMAM facilities

Figure 5 shows findings from data verification relating to RUTF and compares numbers of RUTF cartons consumed recorded in the weekly tallies with the number of cartons recounted from the OTP cards for July 2017. Discrepancies were 10% or more for eight of the nine facilities visited, and greater than 30% in five facilities, with a maximum value of 52%.

#### Explanations for patterns identified

Where discrepancies existed for admissions, total exits and RUTF consumption, the reported values tended to be higher than the recounted values (see Figs. 3, 4 and 5). This suggests either that admissions and exits were over-reported and/or that OTP cards were lost. It is plausible that both factors contribute, since our observations indicated a lack of formal modus operandi for the tallying procedure and for storage of OTP cards, as well as lack of dedicated storage space for the cards. The large size of discrepancies for some facilities perhaps indicates the loss of cards as a major explanatory factor at least for those facilities. Also, given the evidence for misappropriation of RUTF elsewhere in northern Nigeria, this must be mentioned as a possible reason for discarding records in the facilities.

**Fig. 6:**
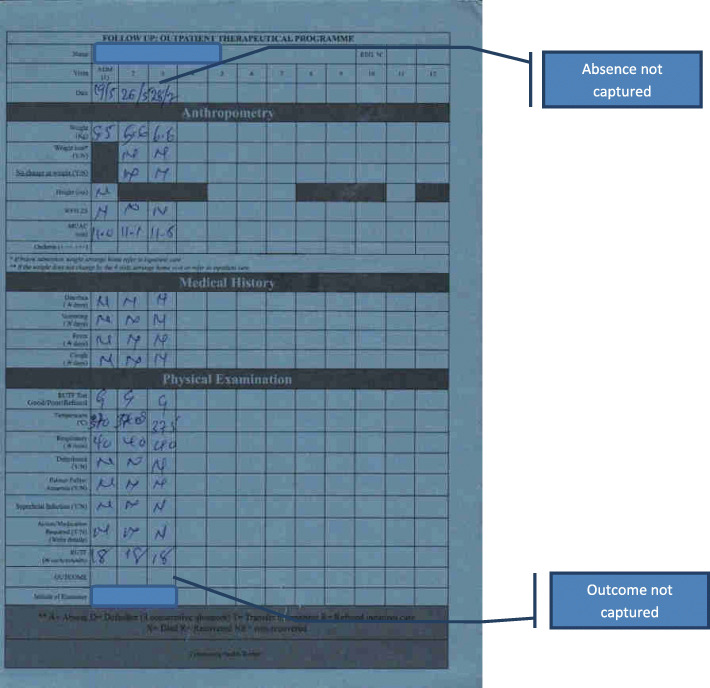
Example extract from OTP card with error in completion

With respect to the underestimates of defaulting and death rates, there are several contributory factors. During facility visits we observed a lack of consistent observance of the protocols described in the national CMAM guidelines [17]: the child should be discharged as a defaulter on their third consecutive absence. Our data verification revealed that absences are often not noted on the OTP cards of absentee children, and even after three absences their cards are commonly kept “active” and stored together with those for children attending. For example, Fig. 6 shows an OTP card for a child that between 26/5 to 28/7 should have been recorded as absent and then discharged. Also, the outcome cell on OTP cards is often left blank, as in the card in Fig. 6, so that during recounts it is not clear in which week and using which exit category the staff had noted the child’s exit in the tally. Interviews revealed that CMAM staff may rely on information from clients or, occasionally, community volunteers to identify defaulters, and other children who do not return after a long absence are classified as “non-recovered”.

Observations and interviews indicate two main underlying explanations for the inconsistencies between CMAM operations and national guidelines, which affect accuracy and reliability of the CMAM data:

- Firstly, the CMAM programme (like the wider government health service) is affected by severe resource constraints. Due to this there is inadequate provision of forms, electricity and storage; and inadequate human resources, high workloads and capacity issues. An NFP commented “*It would be helpful to have a computer because then I wouldn’t need to drive to the state office to submit my data - other units at LGA level have them*”. Expertise is frequently lost – a UNICEF officer commented “*CMAM-experienced staff are often transferred away to non-CMAM sites and this impacts on quality of service. So we request that if staff must be transferred, they be moved to another CMAM site”*. All these constraints on resources inevitably affect the quality of services and monitoring data.
- Secondly, although CMAM trainings were reported to include sessions on how to complete forms and tally data; there is a lack of clear printed guidelines, training materials and protocols on CMAM data capture for health-workers. The national CMAM guidelines were not at hand in any of the facilities visited; and in any case do not include details on data tallying, protocols for storing paper records, or the new SMS system. Also, we observed that various versions of forms for recording data are being used.

### Data accuracy and reliability: findings from secondary data analysis of LGA and state level data

We compared the weekly tallies collected at health facilities (the source data for both the SMS- and paper-based datasets, see Fig. 1) with data records at the two next levels of the paper-based monitoring system as follows: (a) data from the paper records held at LGA level, where tallies are consolidated by the NFP for all facilities in the LGA; and (b) the electronic datafile generated at state-level from the paper records submitted by the NFPs. We did so in order to assess whether discrepancies are introduced into the paper-based dataset when data are aggregated in LGA offices, and/or when they are entered into the spreadsheets by personnel at state level.

Some discrepancies between the weekly paper tallies and the LGA reports were noted for admissions (− 35 and 12%) and exits (12 and 2%) for two facilities. The values entered at LGA level carried over into the paper-based datafile indicating data transfer between facility and LGA level as a potential source of inaccuracy in the electronic data.

The analysis found very few errors in data entry at state level: The state-level electronic datafile matched the LGA paper-based data for exits and admissions in all but one instance.

#### Explanations for patterns identified

The discrepancies observed between the LGA reports and weekly tallies could be due to introduction of errors when the NFP copied data between paper forms. Alternatively, errors may have been introduced when the facility-in-charge copied data from the paper form stored at the facility (viewed by the study team) and the paper form they later submitted to the NFP. Both factors could plausibly contribute to the discrepancies, since our observations indicated a lack of formal quality assurance procedure for data transfer between levels.

### Data accuracy and reliability: findings from secondary data analysis of federal level data

We compared the electronic datafiles from the paper-based and SMS systems for all records between January and July 2017 (*n*=299 facility-months). The datasets should be consistent since they are both derived from the same paper records (OTP cards and weekly tallies) held at the facilities. For those 46 facilities for which information was available, Table 2 shows the proportion of records where the data on CMAM outcomes were different in the two data systems. The differences between the data values do not have a consistent direction.

**Table 2:** Proportion of months for which reported outcomes differed between SMS and paper-based systems

	New admissions	Cures	Defaulters	Deaths	RUTF consumed
Proportion of facility-months where Paper = SMS	66%	58%	78%	87%	36%
Proportion of facility-months where difference >=10%	23%	30%	22%	13%	41%
Proportion of facility-months where difference < 10%	11%	12%	0%	0%	23%
Total	100%	100%	100%	100%	100%

#### Explanations for patterns identified

It is somewhat surprising that there is not a closer match between the paper-based and SMS datasets, given that the source data (paper tallies) are the same. A potential source of discrepancies is errors introduced during data entry to mobile phones. Although no such errors were observed during the study, the study team visited only nine facilities, and the presence of observers may have positively affected conduct of the data entry to mobile phones on those few occasions, while the data in Table 2 are derived from 46 facilities over 7 months. There is no formal quality assurance procedure for the process of sending weekly data by SMS, so errors in the SMS dataset are plausibly introduced at this stage. Also, as noted above, errors in the paper dataset are introduced when data from the facilities are aggregated at LGA level.

### Findings pertaining to completeness and timeliness from secondary data analysis

**Fig. 7:**
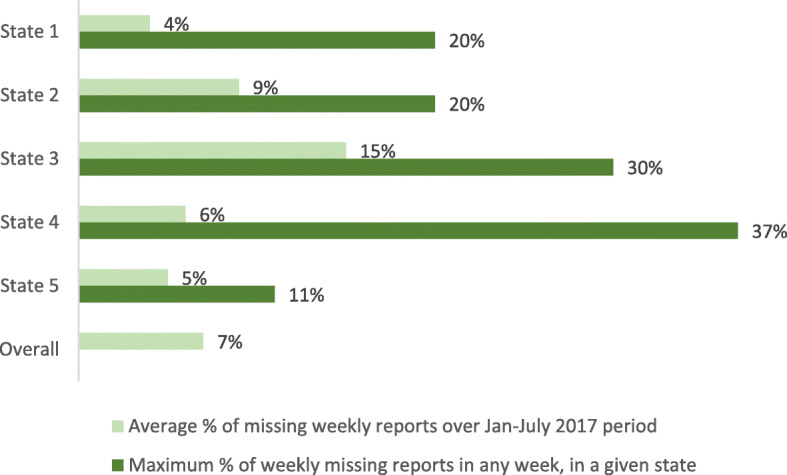
Proportion of weekly reports missing across 7 months in each state

For the paper-based system, all facilities reported on a monthly basis throughout the reference period, so reporting was complete in terms of months with valid observations. In contrast, completeness of the SMS dataset was deficient with respect to weeks with valid observations. We found that, on average, 7% of weekly reports were missing (with a maximum of 37% missing reports from one facility as shown in Fig. 7).

Both the paper and SMS data had relatively good completeness with respect to missing values in specific variables. For example, for the SMS dataset, key reporting variables (admissions, exits) were missing for only 0.1% of weekly reports, and RUTF stock reported at the beginning of the week was missing for 5.9% of weekly reports. For the paper dataset, the numbers of children in treatment at beginning and end of month were missing for 11 and 12% of monthly reports respectively.

**Fig. 8:**
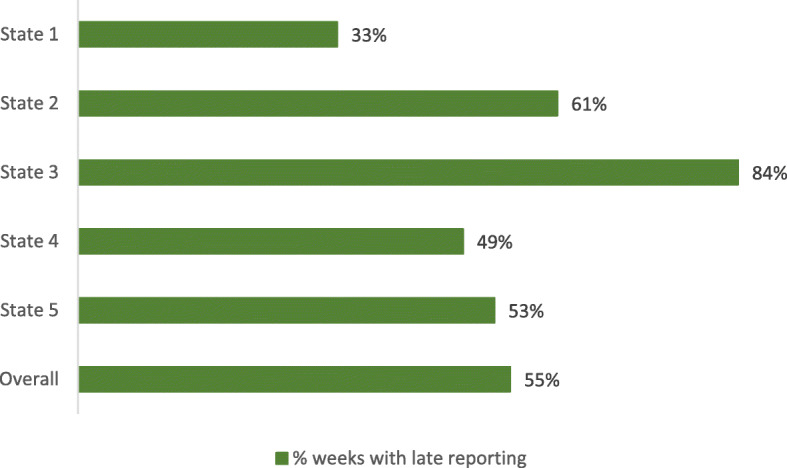
Proportion of weeks with late reporting across 7 months in each state

The timeliness of the SMS data was weak. We assessed this using the indicators produced within the UNICEF dashboard, which allows facilities to report until the Monday following the OTP day. Figure 8 shows that data for slightly more than half of the weeks reported came in late for the period of weeks 1 to 30 (January to July) 2017.

#### Explanations for patterns identified

The missing values and inconsistencies noted during secondary data analysis appeared to be affected by the absence of a formal process to verify the numbers of children in the programme, based on child-level records. A key challenge was that staff carry over the number of “children in treatment” from the end of the previous week to the start of the new week, rather than by counting OTP cards. The practice of carrying over data is also used for the paper dataset (data are carried over from the end of previous month). The variable “number of children in treatment” is used to calculate requirements for RUTF, so this is a potential incentive for maintaining inflated values in the dataset.

With respect to the relatively poor timeliness of weekly report submission and incompleteness of the resultant SMS dataset revealed by our secondary data analysis, the coverage and reliability of the mobile phone network was perceived to be the major challenge. For example, one recently appointed CMAM-in-charge (whose CMAM clinic takes place on Mondays, and whose friend X is also a CMAM-in-charge) said.

“*I’m expected to send the SMS report the same day as the CMAM clinic, but can’t always do this because of the phone network. Last week I couldn’t send it until Friday, and so contacted X who said it was a problem for everyone, and that I should just keep trying.*”

While most of Nigeria has good network coverage, there are pockets where coverage is lacking, and also network quality varies across time and space. Another factor may be low motivation to submit the texts. While study interviews revealed health-workers’ high motivation to submit their data on time, and frustration when this was not possible, these sentiments may not be ubiquitous. The paper-based CMAM monitoring is part of the formal governmental reporting system, while the parallel SMS-based monitoring may be considered less important as it is not owned by the health system.

## Discussion

For our assessment of the SMS and paper-based CMAM monitoring systems in northern Nigeria, we have summarised the major findings relating to accuracy, reliability, completeness and timeliness. The study’s primary data collection is based on a subset of purposively selected facilities and the findings can therefore not be generalised to the wider CMAM programme in terms of the values of indicators calculated. However, we deliberately chose to undertake a small-scale contextualised study in a single state, and used mixed qualitative and quantitative methods, because we needed to understand the origins of any identified gaps in data quality. We analysed the full system including inputs and processes, and hence contend that strengths, weaknesses and patterns detected are very likely to be found across the CMAM programme, as they relate to the way the system was designed and is operated, in the facilities where observations took place and elsewhere. In this section, we discuss the implications of the most salient findings to the CMAM programme in Nigeria and more widely.

### Discrepancies between recounted data from OTP cards and data from the paper records held by facilities

We found discrepancies between recounted and reported data for admissions, total exits, defaults and deaths, and RUTF consumption. It is not possible to distinguish between loss/removal of OTP cards and errors during data tallying as explanatory factors, but the large size of discrepancies for some facilities indicate loss or discarding of cards as the major factor at least for those facilities. This highlights the importance of developing quality assurance processes for the tallying procedure, as well as guidance on the storage of paper records. Without improvement of storage practices, attempts to audit data quality will remain a challenge.

### Artificially low default rates and difficulty distinguishing death/default

The discrepancies in the reported and recounted “death or default” rates highlight several concerns. Firstly, although international guidelines state that children should be followed up periodically after discharge [4], this does not occur in in the sampled state due to the absence of incentives available to compensate community volunteers. Without a functioning system of home visits, it is impossible to know if a defaulted beneficiary is alive and thus a ‘true’ defaulter, or if they have died. This concern may be relevant to other countries where the system for community-level follow up is weak, since it raises questions about the possibility of accuracy in the reporting of default and death rates.

Secondly, our data point to artificially low death and/or default rates associated with the CMAM programme in Nigeria. The explanatory factors relate mainly to discrepancies between actual treatment practices and national CMAM guidelines [17]. Although the guidelines include a clear criterium for defining defaulters, that of three consecutive absences, such breaks in treatment are often not noted on the OTP cards of absentee children, and the children are not discharged. The CMAM guidelines do not describe a standard process for identifying defaulters each week by looking at the OTP cards of children who have not attended, and our observations confirmed that this process does not occur in a systematic way. Instead, CMAM staff may rely on information from clients or, occasionally, community volunteers to identify defaulters.

Our findings highlight the importance of instigating a system to identify defaulters through examination of OTP cards, as part of a quality assurance process of the weekly tallying procedure. With respect to deaths, since for many facilities it is currently impossible to record an accurate measure, it seems logical to propose dropping the indicator of mortality from the data recording process. However, death is a valuable outcome measure, so it would be preferable to work towards strengthening the community-based component of the programme, and meanwhile instigate a system whereby the indicator is flagged as unreliable for those CMAM facilities without community volunteers.

The importance of these proposals relates to the common use of death, defaulting and recovery rates as indicators of the effectiveness of CMAM programmes, as defined by Sphere Standard 2.2 relating to management of SAM [26]. This standard defines that the proportion of discharges from therapeutic care who have died, recovered and defaulted should be < 10, > 75 and < 15% respectively. Our findings suggest that the performance indicators of defaulting and death reported from the Nigeria system are likely to be under-estimates and provide a misleadingly good impression of the programme’s performance.

### An important source of error currently undetectable through data verification

We noted that data recorded for the variable “children in treatment” for the beginning of each period (week/month) are carried over from the end of the previous period (week/month). We suggest that recording of number of children in the programme should be verified each week based on child-level records and be reported through the information system. This would enable cross-checks of amount of RUTF distributed against number of children attending, and identification of facilities for closer scrutiny.

This suggestion is not made lightly, since any increase in data collection and reporting requirements would be an additional burden to staff who are already over-worked. Also, it requires a change to the current approach and a protocol would have to be developed and tested. But because inaccuracies in the indicator affect allocation of RUTF, it is important to tackle this issue.

### The current need for both SMS and paper-based system

There are several advantages of the SMS reporting system over the paper-based system. The most obvious are the fewer intermediate data transfer and processing steps (which increase the risk of introducing errors in the data), and that it is not necessary to physically transport records. In the SMS system, data reach the federal level faster, which facilitates more rapid programme decision-making, even considering the study findings on delays in submission of data. Interviews revealed that RUTF stock alerts are transmitted automatically when low levels of stock are detected within the dataset. There is also a reduced risk of data loss that may arise from loss or destruction of paper forms.

The drawbacks of the SMS system are that sending the texts adds an extra task to the health-workers’ workload, that the completeness and timeliness of the dataset is dependent on network coverage, and that the system is not institutionalised. Remote facilities are particularly affected by poor network coverage, which can delay transmission of the data. Further, if the SMS system were to operate without the paper-system in parallel, the LGA and state levels would be less aware of data reported by facilities under their supervision. This brings the associated risk of reducing quality assurance mechanisms at those levels, as well as motivation to use the data.

We construe that in the current circumstances, it is not possible to phase out the paper-based system for transferring data. This would require a more reliable network coverage, and a means of sharing the SMS system findings with LGA and facility level staff. The latter might be done via paper reports, or a “real-time” report that could be accessed on mobile phones (in addition to the existing practice of discussing the data at state-level meetings). Another action that would likely improve motivation and thereby data quality is institutionalisation of the monitoring system, by inclusion of the nutrition indicators in the routine health information system.

## Conclusions

This study identified that implementation of a mobile technology for CMAM data transfer in Nigeria benefits the programme by providing rapid electronic notification of stock shortages. However, mobile network coverage is not sufficiently reliable for the SMS system to replace the paper-based system. Several of our findings concur with those of earlier studies which indicated that national protocols for admissions, and definitions of defaulting and recovery, were not being applied; and that outcome indicators and counts of children admitted were inaccurate [13–16]. These findings are significant because they indicate the performance indicators of defaulting and death reported from the Nigeria system are under-estimates, and provide a misleadingly good impression of programme performance. Since a major underlying explanation for the observed inaccuracies are the severe resource constraints under which the programme operates, gaps between reported and actual performance might be inferred for CMAM programmes in other countries with under-resourced health systems. We also found the current clinic-based system for CMAM treatment in northern Nigeria is ineffectual at detecting child deaths at home. This challenge raises questions about the possibility of accurate death and default rates in other countries with weak home follow-up systems.

The study highlights the need for more comprehensive training, supervision and quality assurance focused on the correct completion of OTP forms, weekly tallying and data transfer. A crucial step is recognition and acceptance that if data quality improves, performance indicators may get worse. Temporary deterioration in performance indicators can be a positive sign of better data processes and reporting, rather than a sign of a decline in performance.

## Supplementary information

**Additional file 1.** CMAM Interview and verification instruments. Facility-level data collection tools; LGA-level data collection tools; State-level data collection tools; Federal-level data collection tools; Protocol for verification of CMAM admission and discharge data; OTP data verification collection forms, and RUTF data verification collection forms.

## Data Availability

The quantitative datasets generated and analysed during the current study are currently not publicly available. The CMAM management systems information data are held by UNICEF and the Government of Nigeria. For access to these data contact Dominic Elue, Deputy Director, Head of Nutrition Information System, Federal Ministry of Health, Nigeria rhodome@yahoo.com . Anonymised data from OTP card recounts are available from the corresponding author on reasonable request.

## Abbreviations

CIFF: Children’s Investment Fund Foundation
CMAM: Community-based management of acute malnutrition
LGA: Local Government Area
MoH: Ministry of Health
NFP: Nutrition Focal Person
OTP: Outpatient therapeutic programme
OPM: Oxford Policy Management
SAM: Severe acute malnutrition
SMS: Short message service
SNO: State Nutrition Officer
