# Supplementary material for: Assessment of monitoring systems in the management of severe acute malnutrition in northern Nigeria
Source: BMC Nutr. 2021 Jan 12;7:2. doi: 10.1186/s40795-020-00405-z (PMC7802179; doi:10.1186/s40795-020-00405-z)
Supplement: Supplementary file 1 — Additional file 1. CMAM Interview and verification instruments. Facility-level data collection tools; LGA-level data collection tools; State-level data collection tools; Federal-level data collection tools; Protocol for verification of CMAM admission and discharge data; OTP data verification collection forms, and RUTF data verification collection forms. [file 40795_2020_405_MOESM1_ESM.pdf]

FACILITY LEVEL

Activities at facility level are likely to be undertaken by a range of staff. Using the responses from the first tool, please interview or observe the relevant staff for each activity, and note their name and job title each time. Please use the question numbers when writing notes on separate sheets during the interviews.

Introduction: *Explain why we are here, for example "We are finding out how the system works for collecting, storing and transferring CMAM programme data. We are independent, and not part of UNICEF or the government. We are hoping that by interviewing and observing how things happen at the facility, we will discover ways the system can be improved."*

TOOL F1 Interview with Head Nurse or CMAM-in-charge at the facility

Please write notes on the responses using separate sheets, using the question numbers on the left.

F1.1 Your role

- How long have you been in this post? Can you please explain your role?
  - *If interviewing head nurse:* To what extent are you involved with the operation of the CMAM programme? *Probe for approximate proportion of time spent*
  - *If interviewing CMAM in charge:* What are your responsibilities / tasks in relation to the CMAM programme?

F1.2 CMAM programme operation

- How many health workers and volunteers provide the CMAM services at this facility?
- We would like to talk to them later, so please tell me their names and what they are responsible for.
  On the separate sheet, list names, job titles and tasks. If these tasks are not mentioned, ask about: storing cards and reports, tallying data, and data transfer.
- When the programme is busy, do you sometimes get help from other staff?
  If yes, list their positions, and the tasks they are responsible for when they assist.
- Do you sometimes get extra help from outside this facility? *If yes, give details.*

F1.3 Supervision that you do

- Who do you supervise within the CMAM programme?
  On the separate sheet, list the job titles and tasks of those who are supervised by the respondent.
- How is the supervision organised?

F1.4 Your training

- What is your level of education?
- When were you last trained in CMAM-related tasks, and what training were you given? *(probe for training in SMS data transmission if this is not mentioned)*
- How often do you get training in CMAM-related tasks?

	• Is there anything you were trained to do that is difficult or impossible, if so what and why?<br><br>• Does the position of (head nurse/ CMAM-in-charge) require you to do anything that you were not trained for?<br><br>• What changes in the training would you like to see, that will help you do your job well?
**F1.5 Supervision of your work**	
	• Who supervises or checks your work for CMAM?<br><br>• How is the supervision organised?<br><br>• What is your opinion about the supervision?<br><br>• Is there anything you need to do in CMAM for which you do not have sufficient supervision or guidance (for example, protocols, standing orders, Job Aids, or guidelines) to do well?<br><br>• What changes in support would you like to see, to meet your supervision and guidance needs?
**F1.6 Resources**	
	• Do you have enough time to conduct high quality service delivery?<br><br>*Probe for which CMAM or other tasks would benefit if the respondent had more time available.*<br><br>• Has the introduction of the SMS system for CMAM changed your workload? If yes, how?<br><br>• Do your colleagues and management think it's important to have good systems to collect CMAM data and transfer CMAM data from the facility to higher levels?<br><br>• Do you follow written guidelines/protocols? If so, please can you show them to us?<br>*Probe for Nigeria CMAM guidelines, training material, USAID guidelines*<br><br>• What additional resources would help you do your job related to CMAM better?

TOOL F2 Checklist/ observations on storage of CMAM data at facility level

**Name of Facility:**<br>**Name of person/people observed:**<br>*Please write notes in the spaces below each question. If you need more space, continue on a separate sheet using the question numbers on the left.*		**Date of visit:**<br>**Observer:**	
**F2.1**	Where are OTP cards stored for current attendees?<br>*Ask to see a card for a child admitted to the programme one week ago.*<br>*If there were no admissions in the past week, ask to see the most recent admission card.*		
	Comment how easy it was to find the card, and what system is used for filing/ storing the cards		
**F2.2**	Are the current OTP cards locked up when the facility is closed?		**YES/ NO**
**IF YES**	**F2.3**	Who has access to the key?	
		Name and job title	
**F2.4**	Where are the OTP cards stored for past attendees?<br>*Ask to see a card from a child admitted to the programme during the first week of July 2017*		
	Comment on filing system used, and how easy it was to find this card		
**F2.5**	Are the cards for past attendees locked up when the facility is closed?		**YES/ NO**
**IF YES**	**F2.6**	Who has access to the key?	
		Name and job title	
**F2.7**	For how long do you keep the cards? *Ask to see the oldest card stored*		
	Date of first visit on the oldest card		
**F2.8**	Is there written guidance on the length of time you should keep the cards?<br>*IF YES, ask to see the guidance, and note the content below.*<br>*IF NO,* Have you had verbal guidance?<br>*If yes, summarise the verbal guidance below.*		**YES/ NO**<br>**YES/NO /n.a.**
	Guidance		
**F2.9**	Is there written guidance on methods of storing the cards so they remain confidential? *IF YES, ask to see the guidance, and note the content below.*<br>*IF NO* Have you had verbal guidance?<br>*If yes, summarise the verbal guidance below.*		**YES/ NO**<br>**YES/NO /n.a.**

	Guidance	
**F2.10**	How do you dispose of the cards?	
	Disposal method	
**F2.11**	Are there other places you keep records about the individual children?<br>*Probe for registration book, RUTF cards*<br><br>If yes, where are these records stored, and are they kept locked up?	**YES/ NO**
	Details of other records and where they are stored	

TOOL F3 Checklist / observations on tallying weekly facility CMAM data

Name of Facility: **Date of visit:**
Name of person/people observed: **Observer:**

Please write notes in the spaces below each question. If you need more space, continue on a separate sheet using the question numbers on the left.

**F3.1**	When do you do the aggregation (combining) of individual data into weekly totals?<br>*Circle one of the responses in the column on the right.*	**At end of CMAM day / Another day that same week / End of month**
**F3.2**	Have you changed how, or how often, you do this since the SMS system for data transfer was introduced? If yes, please explain	**YES/ NO**
	Change in process	
**F3.3**	Do you do tally the data together with someone else?<br>If yes, who do you do this task with?	**YES / NO / It varies**
	Name and job title	
**F3.4**	Do you have difficulty finding time for this task? Please explain	**YES/ NO**
	Difficulties	
**F3.5**	Can you show me how you do the tallying? *(or observe later)*	
	Comments	
**F3.6**	Do you find the tally form easy to use? Please explain	**YES / NO**
	Ease of use	
**F3.7**	Do you check the tallying was done correctly?<br>If so what method do you use? Can you show me?	**YES/NO**
	Method	
**F3.8**	Do you ever run out of the necessary forms for data collection and tallying?	**OTP cards YES /NO**<br>**Tally sheet YES/NO**
	Note the last time there were no forms, and what type of forms	

TOOL F4 Checklist /observations on storing aggregated CMAM data at facility level

<table>
<tr><td colspan="4">Name of Facility: Date of visit:
Name of person/people observed: Observer:

Please write notes in the spaces below each question. If you need more space, continue on a separate sheet using the question numbers on the left.</td></tr>
<tr><td>F4.1</td><td colspan="3">Where do you keep copies of the weekly / monthly tally reports?
Ask to see the report from the first week of July 2017</td></tr>
<tr><td></td><td colspan="3">Comment on ease of retrieval</td></tr>
<tr><td>F4.2</td><td colspan="2">Are the records locked up when the facility is closed?</td><td>YES/ NO</td></tr>
<tr><td>IF YES</td><td>F4.3</td><td colspan="2">Who has access to the key?</td></tr>
<tr><td></td><td></td><td colspan="2">Name and job title</td></tr>
<tr><td>F4.4</td><td colspan="3">For how long do you keep the reports?
Ask to see the oldest report stored</td></tr>
<tr><td></td><td colspan="3">Note the month and year of the oldest report</td></tr>
<tr><td>F4.5</td><td colspan="2">Is there written guidance on the length of time you should keep the reports? IF YES, ask to see the guidance, and note the content below.
IF NO Have you had verbal guidance?
If yes, summarise the verbal guidance below.</td><td>YES/ NO

YES/NO /n.a.</td></tr>
<tr><td></td><td colspan="3">Guidance</td></tr>
<tr><td>F4.6</td><td colspan="2">Is there written guidance on methods of storing the reports so they remain confidential? IF YES, ask to see the guidance, and note the content below.
IF NO Have you had verbal guidance?
If yes, summarise the verbal guidance below.</td><td>YES/ NO

YES/NO /n.a.</td></tr>
<tr><td></td><td colspan="3">Guidance</td></tr>
<tr><td>F4.7</td><td colspan="3">How do you dispose of the reports?</td></tr>
<tr><td></td><td colspan="3">Disposal method</td></tr>
</table>

TOOL F5 Interview on transfer of CMAM data from the facility

Please write notes on the responses using separate sheets, and using the question numbers on the left.

F5.1 General information about the pen-and-paper system, and the SMS system

- Describe the system for transferring weekly, or monthly paper reports to LGA level or state level.
- How often do you do this?
- Describe the system for sending the weekly aggregated facility data to federal level using SMS.
- How often do you send the texts?
- Do you send the texts with someone else present?
- Can anyone else do this task instead of you?
- Is the SMS system user-friendly? *Ask for demonstration and record your own opinion too.*

F5.2 Training and supervision for the SMS system

- When were you trained in the SMS system?
- When were the other person/people you mentioned above trained?
- What support is provided for data transmission? (compare paper system with SMS system)
- What are the different challenges faced in data transmission with the two systems?

F5.3 Timeliness for sending paper reports

- Do you keep a record of the date you sent weekly, or monthly, aggregated facility reports on paper to LGA level or state level?
- *Note down the dates that paper reports were sent for the three months May - July 2017.*
- Is there a deadline for submission of the facility monthly report on paper? If yes, what is it?

F5.4 Timeliness for sending SMS reports

- Do you keep a record of transmitting the weekly aggregated report to federal level, with date?
  If yes, note down the dates that reports were sent between May and July 2017.
- Is there a deadline for submission of the facility weekly report to federal level by SMS?
  If yes, what is it?

F5.5 Comparison of the systems

- What are the advantages or disadvantages of the new SMS system compared to the paper-based system?
- In your view, what is the greatest advantage, and the greatest disadvantage, of the SMS system compared to the paper-based system?
- Are there benefits of having both systems running at the same time?

TOOL F6 Checklist on supervision and quality assurance of the CMAM programme at facility level

**Name of Facility:**<br>**Name of person/people observed:**<br>*Please write notes in the spaces below each question. If you need more space, continue on a separate sheet using the question numbers on the left.*	**Date of visit:**<br>**Observer:**	
**F6.1**	How many times did you receive a supervisory visit during the last three months? *If no visits, move straight onto question 6.10*	**No visits / 1 / 2 / 3 / 4 or more**
**F6.2**	Who made the last three supervisory visits?<br>*Circle those that apply on the right (If possible, later record the visit dates, and names + institutions of those who made visits in the last 3 months)*	**(1) LGA nutrition focal point**<br>**(2) State nutrition officer**<br>**(3) Other……………………**
For the next questions, the numbers (1) to (3) refer to the visits specified in question 6.2		
**F6.3**	Did the supervisor discuss the performance of the CMAM programme when he/she visited your facility?	**(1) YES/ NO**<br>**(2) YES/ NO**<br>**(3) YES/ NO**
**F6.4**	Did the supervisor help you make a decision based on the CMAM information when he/she visited your facility?	**(1) YES/ NO**<br>**(2) YES/ NO**<br>**(3) YES/ NO**
**F6.5**	Did the supervisors leave a record of their visit with comments, at the time of their visit?<br><br>*If yes, check the visit record, and note you have seen it on the right*	**(1) YES, observed/ NO**<br>**(2) YES, observed/ NO**<br>**(3) YES, observed/ NO**
**F6.6**	Did the supervisors send a report/ feedback/ note some days or weeks after the last supervisory visits?<br><br>*If yes, check the feedback, and note you have seen it on the right*	**(1) YES, observed/ NO**<br>**(2) YES, observed/ NO**<br>**(3) YES, observed/ NO**
**F6.7**	During the last three months did you receive instruction(s) from the facility-in-charge, or the LGA office, to:<br>• Check the accuracy of data?<br>• Fill the monthly report form completely?<br>• Submit the report by the specified deadline?<br>*If yes, check the instruction(s) and note you have seen it on the right*<br><br>IF NO, have you ever had an instruction like this? *If yes note it below*	**YES, Observed /NO**<br>**YES, Observed /NO**<br>**YES, Observed /NO**
	Instruction	
**F6.10**	What are the main challenges you face in your role with respect to making sure the CMAM data are of good quality (meaning the data are ready by the deadline, they are complete and they are true)?<br>*Probe for capability, confidence, time, etc.*	
	Challenges	

TOOL F7 Checklist about CMAM data use at facility level

**Name of Facility:**<br>**Name of person/people observed:**<br>*Please write notes in the spaces below each question. If you need more space, continue on a separate sheet using the question numbers on the left.*	**Date of visit:**<br>**Observer:**	
**F7.1**	Does the facility issue any report(s) containing CMAM data/ information?<br>*If yes, record details below of each report*	**YES Observed /NO**
	Report title ***Frequency/ year*** ***Number of reports issues in past year***	
**F7.2**	Does the facility hold routine meetings to review the CMAM programme?<br>Does the facility hold routine meetings to review general managerial or administrative matters? *Ask the following questions about the CMAM meeting, or if there is no CMAM meeting, ask about the general one:*<br>• Are you invited to contribute to the meeting?<br>• How frequently is the meeting supposed to take place?<br>• How many times did the meeting take place in the last three months?<br>• Are official records of management meetings kept?<br>*If yes, check the record for the last meeting and note below which, if any CMAM-related issues were discussed*	**YES/NO**<br>**YES/NO**<br><br>**YES/NO**<br>**Weekly/ fortnightly /monthly/quarterly /ad hoc / n.a.**<br><br>**7–12 times / 3- 6 times / once or twice /never /n.a.**<br><br>**YES/NO**
	Date of meeting and issues discussed	
**F7.3**	Do you participate in meetings at LGA level to discuss CMAM performance?<br>If yes, did you participate in one during the last three months?	**YES/NO**<br>**YES/NO**
**F7.4**	Please describe examples of how the facility uses CMAM data<br>*(if no examples are given, record NO EXAMPLES GIVEN, but then prompt to check if the CMAM data are used to forecast needs for training, supervision, supplies including RUTF)*	
	Details of data use	
**F7.5**	Would you like to use the CMAM data more, and if so, what are the challenges you face in doing so?	
	Challenges *(probe capability, confidence, time)*	
**F7.6**	Are findings from analysis of CMAM data done at state or federal level shared with you? **YES/NO**<br>• If NO, would it be useful for you to see findings from the LGA, State, or whole programme? What indicators (e.g. admissions, cure rates) What would you use them for?<br>• If YES, what findings are provided to you? Do you use them, and if so, what for?	
	Data that would be useful, and potential use OR Data currently provided and actual use + Comments	
**F7.7**	Does the facility display any CMAM data? *If yes, indicate types of data displayed below, and if data are updated for last reporting period on right.*<br>Table **YES/NO** Data:<br>Graph/Chart **YES/NO** Data:<br>Map **YES/NO** Data:	**YES/NO**<br><br>Updated? **YES/NO**<br>Updated? **YES/NO**<br>Updated? **YES/NO**

Tool F8: Interview with CMAM health workers (not the volunteers)

Please write notes on the responses using separate sheets, using the question numbers on the left.

F8.1. Your role

- How long have you been in this post?
- Can you please explain your role?

F8.2. CMAM programme operation

Using the OTP form, please briefly explain (we will later observe this process):

- What happens when clients enter the CMAM programme?
- Who is RUTF given to?
- Is there a manual or checklist to remind you what to do? If so, please show us any written guidelines that you follow

F8.3 Your training

- What is your level of education?
- When were you last trained in CMAM?
- What training were you given?
- Is there anything you were trained to do in CMAM that is difficult or impossible, if so what and why?
- Is there anything you are required to do when carrying out CMAM that you were not trained for?
- What changes in the training would you like to see, that will help you do your job well?

F8.4. Supervision of your work

- Who supervises your work for CMAM?
- How is the supervision organised?
- When were you last supervised?
- What is your opinion about the supervision?
- Is there anything you need to do in CMAM for which you do not have sufficient guidance or supervision to do well?
- What changes in support would you like to see, to meet your supervision and guidance needs?

F8.5 Recording and storing CMAM data from individuals

- What kind of forms do you use during the visits?
- Is the OTP form easy to use?
- Does the OTP form have enough space to record data?
- Does the OTP form take a lot of time to complete?
- What do you do with the OTP cards at the end of the day?
- Please explain to us and show us on the OTP card how you identify children and record information for children
  - who have returned after having been absent for more than three weeks
  - who have returned within two months of being discharged
  - who are referred from stabilisation care or transferred from another OTP site

F8.6 CMAM data quality

- What are the challenges you face when working in the CMAM programme?
- Does it ever happen that necessary information is not recorded, or recorded incorrectly?
- If so, how could the programme be changed to avoid this happening?

Tool F9: Checklist /observations during operation of the CMAM programme at the facility

Please try to observe at least five children of which three are new admissions. For each child, observe the process of triage and admission and check the relevant boxes. If the child is an existing case, circle n.a. where necessary. It is likely that these questions will not be answered in the order in which they are included below. Please try to answer as many as possible, and ***make notes on a separate sheet using the questions numbers on the left about your observations*** *of the processes followed.*

**Name of Facility:**<br>**Name of person/people observed:**		**Date of visit:**<br>**Observer:**
**F9.1 Case type**		
	Is this child a current case or a new admission today?<br><br>If the child is a new admission, what type?<br>*Circle one of the six options on the right once the type of admission becomes clear to you.*<br>*Describe below what questions were asked by the CMAM health worker to establish the type of admission.*<br><br>*If you are not sure about the category, explain why below.*	**Current case / New admission / DK**<br><br>**New case = First visit /**<br>**New case = Relapsed [1]/**<br>**Old case = Referred from stabilisation care/**<br>**Old case = Transferred from other OTP site/**<br>**Old case = Returned defaulter[2]/**<br>**DK**
	Comment	
**F9.2 Recording age**		
	Was age recorded?<br>What age was recorded?<br><br>How was age established if recorded? *Circle one of the three options on the right*	**YES / NO / DK**<br>**Age: ………………… months**<br><br>**Asked mother / Asked to see birth registration or other document / DK**
**F9.3 MUAC measurement**		
	Was a full measuring tape or coloured MUAC tape used?<br><br>Correct process for measurement of MUAC: *Indicate if you observed each of the three standards on the right.*<br><br>What was the measurement? *Enter the actual measurement if a full tape was used, or circle one option if a MUAC tape was used*	**Full measuring tape/ MUAC tape / DK**<br><br>**Arm bent to find mid-point: YES/ NO / DK**<br>**Arm straight for measurement: YES/ NO / DK**<br>**Tape snug but not pinching: YES/ NO / DK**<br><br>**MUAC …………………cm**<br>**or**<br>**< 11.5cm / 11.5-12.5cm/ > 12.5 cm/ DK**
**F9.4 Weight measurement**		
	Correct process for measurement of weight: *Indicate if you observed each of the two processes on the right.*<br><br>What was the measurement?	**Minimal clothes: YES / NO / DK**<br>**Scale zeroed: YES / NO / DK**<br><br>**Weight: ………………… kg**
**F9.5 Height or length measurement**		
	Correct process for measurement of height: *Indicate if you observed each of the two standards on the right.*<br><br>What was the measurement?	**Length measured if < 87cm: YES / NO / DK**<br>**Body straight: YES / NO / DK**<br><br>**Height: ………………… cm**

[1] "Relapsed cases" = Child who returns within two months of being discharged as cured.
[2] "Returned defaulter = Child who has been absent for 3 consecutive visits.

F9.6 Derivation of Weight-for-Height

	Was a chart or table used to assess weight-for-height?<br><br>What was the outcome of the assessment? *Circle one option*	**Chart / Table / DK**<br><br>**<-3Z / -3Z - < -2Z / >-2 Z / DK**

F9.7 Check for oedema

	Was the child checked for oedema?<br><br>Correct process to check for oedema: *Indicate whether you observed each of the four stages on the right*<br><br>What was the outcome of the assessment?<br>*Circle one of the six options on the right*	**YES / NO / DK**<br><br>**Thumb used: YES / NO / DK**<br>**Checked top of feet: YES / NO / DK**<br>**Hold for 3 seconds: YES / NO / DK**<br><br>**None / One foot / Both feet /**<br>**Both feet +legs / Both feet+legs+hands /n.a.**

F9.8 Check for medical complications

	Were questions asked about the child's medical history?<br><br>Checks for medical complications: *Indicate whether you observed each of the four physical checks on the right*	**YES / NO / DK**<br><br>**Temperature: YES/ NO / DK**<br>**Respiration (count one min.): YES / NO/ DK**<br>**Check skin pallor: YES / NO / DK**<br>**Check for skin lesion: YES / NO / DK**

F9.9 Appetite test

	Was the child checked for appetite?<br><br>What was the outcome of the assessment?<br>*Circle one of the three options on the right*	**YES / NO / DK**<br><br>**Eats more than one third sachet RUTF/**<br>**Eats less than one third sachet RUTF/ n.a.**

F9.10 Classification of child

	How was the child classified by the health worker?<br><br>Based on the figure below, was this diagnosis correct?<br>*If NO, explain why your classification differs from the health worker's. If DK, explain your uncertainty*	**SAM/ MAM / NORMAL / DK**<br><br>**YES / NO / DK**
	Comment	

Severe Acute Malnutrition	Moderate Acute Malnutrition	Normal
• MUAC < 11.5 cm (red)<br>• WFH < -3 Z scores<br>• Bilateral pitting oedema	• MUAC > 11.5cm and < 12.5cm (Yellow)<br>• WFH > -3 and < -2 Z scores<br>• No oedema present	• MUAC > 12.5CM (Green)<br>• WFH > - 2 Z Scores<br>• No oedema
Assess for;<br>• Severe oedema +++*<br>• Marasmic kwashiorkor**<br>• medical complications<br>• Appetite for RUTF	Give nutritional advice or refer to SFP if available. See chapter 6.	Praise caregiver and give advice for future nutrition needs to be sustained

F9.11 Admission, referral, or discharge

Depending on the diagnosis, answer questions relating to one of the following four categories

	**New case: SAM**	If the child was diagnosed as SAM, how was the child referred? *Circle one option on the right*<br><br>Was this the correct decision?<br>*(OTP if no medical complications + pass appetite test; Stabilisation if medical complications and/or fails appetite test)*	**Referred to OTP / Referred to stabilisation care / DK**<br><br>**YES / NO / DK**
	**New case: MAM**	If the child was diagnosed as MAM, were they given nutrition advice?	**YES / NO / DK**
	**New case: NORMAL**	If the child was diagnosed as normal, were they praised and given nutrition advice?	**Praise: YES / NO / DK**<br>**Advice: YES/ NO / DK**
	**Existing case**	If the child was an existing case, how was the child diagnosed or discharged? *Circle one option on the right.*<br><br>Was this the correct decision?<br>(*Responding to treatment = gaining weight, increasing MUAC, decreasing oedema, good appetite, no severe medical conditions, regularly attends visits.*<br>*Criteria for "Discharge as cured" are in the table below)*	**Not responding to treatment/ Responding to treatment / Discharge as cured / DK**<br><br>**YES / NO**

Criteria of admission	Criteria of discharge
MUAC < 11.5cm	• Minimum length of stay 8 weeks<br>• MUAC >12.5cm<br>• Sustained weight gain<br>• Clinically well
Bilateral oedema	• MUAC > 12.5cm<br>• No oedema for 2 consecutive visits<br>• Clinically well
WFH <- 3 Z scores (if used)	• MUAC >12.5cm and<br>• WFH > -2 SD for 2 consecutive visits<br>• Clinically well

F9.12 Admission process

**If the child is a new case** and was admitted to the OTP, were the following processes followed?	
Complete OTP card	**YES /NO / Was done earlier / DK**
Register the child in the CMAM registration book	**YES /NO / DK**
Give explanations to caregiver	**YES /NO / DK**
Verify immunisation status and update if necessary Prescribe RUTF	**YES /NO / DK**
Complete Child Health card	**YES /NO / DK**
Give nutrition advice	**YES /NO / DK**
Give appointment for next visit to OTP	**YES /NO / DK**
**If child is a current case**, did health-worker ask caregiver for empty RUTF packets?	**YES /NO / DK**

F9.13 Case Type

	After the consultation, check classification of the case type by the health worker. Does it correspond to your classification in Question F9.1? *If NO, record the health worker's classification below and comment on the possible reason for the difference*	
	Comment	

**Location:**	**Date of interview:**	**Interviewer:**
**Name of interviewee:**	**Job title:**	

Question number	

LGA LEVEL

Introduction: *Explain why we are here, for example "We are finding out how the system works for collecting, storing and transferring CMAM programme data. We are independent, and not part of UNICEF or the government. We are hoping that by interviewing and observing how things happen at all levels, we will discover ways the system can be improved."*

TOOL L1 Interview with LGA nutrition officer / LGA M&E officer

Please write notes on the responses using separate sheets, using the question numbers on the left.	
**L1.1 Your role**	
	• How long have you been in this post? Can you please explain your role?<br>• What are your responsibilities in relation to the CMAM programme?<br>• Approximately what proportion of your time is spent on the CMAM programme?
**L1.2 CMAM programme operation**	
	• How many facilities offer CMAM services in your LGA?<br>• How many health workers and volunteers provide the CMAM services in the LGA?<br>• Do the staff sometimes help at facilities other than their own?<br>• Do you ever get extra help for facilities from state or national level?
**L1.3 Supervision that you do**	
	• How is supervision CMAM activities carried out in the LGA?<br>• How often do you visit facilities, and how often is each facility visited?<br>• Are there other people involved in supervision of CMAM activities at LGA level?<br>o If yes, what are their roles?<br>• What support is provided to you for supervision of CMAM activities at LGA level?<br>• What challenges do you face in supervision of CMAM activities at LGA level?
**L1.4 Your training**	
	• What is your level of education?<br>• When were you last trained in CMAM?<br>• What training were you given?

	• Is there anything you were trained to do that is difficult or impossible, if so why?<br>• Does the position of LGA nutrition officer *(or M & E officer)* require you to do anything that you were not trained for?<br>• What changes in the training would you like to see, that will help you do your job well?
**L1.5 Supervision of your work**	
	• Who supervises your work for CMAM?<br>• How is the supervision organised?<br>• When were you last supervised?<br>• What is your opinion about the supervision?<br>• Is there anything you need to do in CMAM for which you do not have sufficient supervision or guidance to do well?<br>• What changes in support would you like to see, to meet your supervision and guidance needs?
**L1.6 Resources**	
	• Do you have enough time to achieve high quality service delivery?<br>*Probe for which CMAM or other tasks would benefit if the respondent had more time available.*<br>• Has the SMS system for CMAM data changed your role and workload? If yes how?<br>• Do your colleagues and management think it's important to have good systems to collect CMAM data and transfer CMAM data from the facility to higher levels?<br>• Do your colleagues and management value the use of information?<br>• Do you follow written guidelines/protocols? If so please can you show them to us?<br>*Probe for Nigeria CMAM guidelines, training material, USAID guidelines*<br>• What additional resources would help you do your job related to CMAM better?<br>*Probe lack of resources e.g. computers/phones*

TOOL L2 Checklist / observations about CMAM data collection and transfer at LGA level

**Name of LGA:** **Date of visit:**<br>**Name of person/people observed:** **Observer:**<br>*Please write notes in the spaces below each question. If you need more space, continue on a separate sheet using the question numbers on the left.*		
**PAPER SYSTEM**		
**L2.1**	• What is your role in gathering paper reports from facility level? How do you do this, and how often?<br>• Do you integrate the data from all the CMAM facilities in your LGA into an LGA-wide report?	**YES / NO**
	Explain how and when the paper reports are gathered.	
**L2.2**	How many facilities in this LGA are supposed to be submitting paper reports each month?	**No of facilities…………..**
**L2.3**	How many facilities submitted reports for July 2017?<br>*Check the records for July 2017 and note number on the right. If the facilities send in weekly reports, then only count the reports as submitted if all the weekly reports are present.*	**No of reports…………..**
**L2.4**	Is there a deadline for the facilities to submit the CMAM monthly report to LGA level?<br>If YES, what is it?	**YES Deadline ………… days after end of month / NO**
**L2.5**	Do you record the date that you collect or receive the CMAM reports from each facility? *If NO go to question* 3.6	**YES/ NO**
	Check the dates of receipts for July 2017, and complete the tally on the right. (The total number of facilities should be the same as for Q3.3)	**Before deadline …………..**<br>**After deadline ………..**<br>**TOTAL………………..**
**L2.6**	Do you keep a record of transmitting weekly or monthly facility reports to state level, with date?	**YES/NO**
**L2.7**	What is the deadline for submission of the LGA monthly facility reports to state level? (if no deadline, go to Q3.8)	**Deadline = ……………… days after end of the month**
	Check the dates that monthly reports for July 2017, from all facilities, were submitted to state level and complete the tally on the right.	**Before deadline ……………..**<br>**After deadline …………….**<br>**TOTAL……………….**

**SMS SYSTEM**		
**L2.8**	Is there a deadline for submission of the facility weekly report to federal level by SMS? If so, what is it?	**YES Deadline ………… days after end of month / NO**
**L2.9**	When the facilities submit their weekly reports by SMS to federal level, do they also inform the LGA office?	**YES / NO**
	If yes, check the dates that reports were sent for July.<br>*(The total should be at least 4 x the number of facilities noted in Q3.2)*	**Before deadline ………………..**<br>**After deadline …………**<br>**TOTAL……………….**
**L2.10**	What are the advantages or disadvantages of the new SMS system compared to the paper-based system? Challenges of each?	
	Pros and Cons ***Challenges***	

TOOL L3 Checklist/ observations about storing CMAM data at LGA level

**Name of LGA:** **Date of visit:**<br>**Name of person/people observed:** **Observer:**		
Please write notes in the spaces below each question. If you need more space, continue on a separate sheet using the question numbers on the left.		
**DATA STORAGE**		
**L3.1**	Do you keep the weekly and monthly reports from facilities?<br>If NO go to question 3.2<br>*If yes, ask to see the system of storage, and to see the report for a facility from the first week of July 2017.*	**YES/ NO**
	Comment on how easy it was to find the report, and the filing system used for storing the reports	
**L3.2**	Are the facility reports locked up?	**YES/ NO**
**IF YES**	**L3.3** Who has access to the key?	
	Name and job title	
**L3.4**	For how long do you keep the reports?<br>*Ask to see the oldest report, and note the date*	
	Month and year of oldest monthly report stored. Comment how easy it was to find it	
**L3.5**	Is there written guidance on the length of time you should keep the reports? *If yes, ask to see the guidance, and note the content below.*<br>IF NO Have you had verbal guidance?<br>*If yes, summarise the verbal guidance below.*	**YES/ NO**<br>**YES/NO /n.a.**
	Guidance	
**L3.6**	Is there written guidance on methods of storing the reports so they remain confidential? *If yes, ask to see the guidance, and note the content below.*<br>IF NO Have you had verbal guidance?<br>*If yes, summarise the verbal guidance below.*	**YES/ NO**<br>**YES/NO /n.a.**
	Guidance	
**L3.7**	How do you dispose of the reports?	
	Disposal method	

TOOL L4 Checklist about supervision and quality assurance of the CMAM programme at LGA level

**Name of LGA:** **Date of visit:**<br>**Name of person/people observed:** **Observer:**<br><br>*Please write notes in the spaces below each question. If you need more space, continue on a separate sheet using the question numbers on the left.*			
**L4.1**	Do you keep records of your supervisory visits to facilities from LGA level?<br><br>*If yes, check the records and note the dates of visits to facilities from LGA level during the last three months.*<br>*Comment on form of notes kept. Then go to Q4.3*		**YES / NO**
	Dates of visits. Comment on record keeping		
**IF NO**	**L4.2**	Approximately how many supervisory visits to facilities from LGA level took place during the last three months?	**No visits / 1 / 2 / 3 /4 or more**
	Comment		
**L4.3**	After visits, do you ever send feedback to facilities with advice or instructions?		**YES / NO**
	Comment		
**L4.4**	Are you informed if others make supervisory visits to facilities, for example from State level?		**YES/ NO**
**L4.5**	What are the main challenges you face in your role with respect to quality control of CMAM data?<br>*Probe capability, confidence, time*		
	Challenges		
**L4.6**	Compared to other tasks you have, how important is your role in quality control?		
	Priority		

TOOL L5 Checklist about current use of CMAM data at LGA level

**Name of LGA:** **Date of visit:**<br>**Name of person/people observed:** **Observer:**<br>*Please write notes in the spaces below each question. If you need more space, continue on a separate sheet using the question numbers on the left.*		
**L5.1**	Does the LGA office issue any report(s) containing CMAM data/ information?<br>*If yes, record details below of each report*	**YES/NO**
	Report title ***Frequency/ year*** ***Number of reports issued in past year***	
**L5.2**	• Does the LGA health office hold routine meetings to review the CMAM programme?<br><br>• Does the LGA health office hold routine meetings to review general managerial or administrative matters?<br><br>*Ask the following questions about the CMAM meeting, or if there is no CMAM meeting, ask about the general one:*<br>• Are you invited to attend the meeting?<br>• How frequently is the meeting supposed to take place?<br>• How many times did the meeting take place during the last three months?<br>• Are official records of management meetings kept?<br><br>*If yes, check the record for the last meeting and note below which, if any CMAM-related issues were discussed*	**YES/NO**<br><br>**YES/NO**<br><br>**YES/ NO**<br>**Weekly/ After 2 weeks / monthly/ quarterly /no schedule**<br>**7 – 12 times / 3 - 6 times / once or twice / never**<br>**YES/NO**
	Date of meeting and issues discussed	
**L5.3**	Please describe examples of how the LGA uses CMAM data<br>*(if no examples are given, record NO EXAMPLES GIVEN, but then prompt to check if the CMAM data are used to forecast needs for training, supervision, supplies including RUTF)*	
	Details of data use	
**L5.4**	Would you like to use the CMAM data more, and if so, what are the challenges you face in doing so?	
	Challenges *(probe capability, confidence, time, lack of access)*	
**L5.5**	Are findings from analysis of CMAM data done at state or federal level shared with you?<br>If YES, what findings are provided to you and how often?	**YES/NO**
	Description	

**L5.6**	If NO to Q5.5, would it be useful for you to be provided with findings from analysis of CMAM data done at state or federal level? **If NO go to L5.12**	**YES/NO**
	Comments	
**L5.7**	Would you like to have findings from analysis of the CMAM data from the complete programme, or only the findings for this state or LGA?	**Complete programme/ Only this state / Only this LGA**
	Comments	
**L5.8**	Which indicators would be most useful?<br>• Total in programme<br>• New admissions over a defined time period<br>• Cure rates<br>• As many indicators as possible	 **YES/NO**<br>**YES/NO**<br>**YES/NO**<br>**YES/NO**
	Comments	
**L5.9**	Would actual numbers, or summary graphs showing the trends be useful?<br>*Circle response on the right, and add any comments below*	**Exact numbers / Graphs / Both**
	Comments	
**L5.10**	How often should the reports be produced and provided to you? *Circle response on the right, and add any comments below*	**Weekly/ Monthly/ 3 mo. / 6 months/ Year**
	Comments	
**L5.11**	Via which means would be best for you to get access to the reports? *Circle response on the right, and add any comments below*	**Email / Paper copy / Webpage**
	Comments	
**L5.12**	Does the LGA office display any CMAM data? If yes, indicate types of data displayed and if data are updated for last reporting period<br>Table **YES/NO** Data type:.. Updated? **YES/NO**<br>Graph/Chart **YES/NO** Data type:.. Updated? **YES/NO**<br>Map **YES/NO** Data type:.. Updated? **YES/NO**	**YES/NO**

Tool L6 Interviews with potential users of data at LGA level

**Location:** **Date of interview:** **Interviewer:**<br>**Name of interviewee:** **Job title:**<br><br>*Please write notes in the spaces below each question. If you need more space, continue on a separate sheet using the question numbers on the left.*		
**L6.1**	Would you be interested to receive reports of information derived from CMAM programme data collected from facilities in this LGA?<br><br>*(Only for users at State level)* Would you be interested to receive reports of information derived from CMAM programme data collected from all the NE and NW states?	**YES/NO**<br><br>**YES/NO / n.a.**
**L6.2**	If yes, which indicators would be most useful?<br>• Total in programme<br>• New admissions over a defined time period<br>• As many indicators as possible	 **YES/NO**<br>**YES/NO**<br>**YES/NO**
	Comments	
**L6.3**	Would it be useful if the indicators were reported by groups, for example by LGA or State? *Add any comments below*	**YES/ NO**
	Comments	
**L6.4**	Would actual numbers, or summary graphs showing the trends be useful? *Circle response on the right, and add any comments below*	**Exact numbers / Graphs /Both**
	Comments	
**L6.5**	How often should the reports be produced and provided to users? *Circle response on the right, and add any comments below*	**Weekly/ Monthly/ Every 3 mo. / 6 months/ Year**
	Comments	
**L6.6**	Which method would be best for you to get the reports? *Circle response on the right, and add any comments below*	**Email / Paper / Webpage**
	Comments	
**L6.7**	What would you use the information for?	
	Potential use of information	
**L6.8**	***Any other comments***	

**Location:** **Date of interview:** **Interviewer:** **Name of interviewee:** **Job title:**	
Question number	

STATE LEVEL

TOOL S1 Interview with State Nutrition Officer / UNICEF funded Nutrition Consultant

Please write notes on the responses using separate sheets, using the question numbers on the left.

S1.1 Your role

- How long have you been in this post? Can you please explain your role?
- Approximately what proportion of your time is spent on the CMAM programme?

S1.2 CMAM programme operation

- How many facilities offer CMAM services in your state?
- How many health workers and volunteers provide the CMAM services in the state?
- Do the staff sometimes help at facilities other than their own?
- Do you ever get extra help for facilities from national level?

S1.3 Supervision that you do

- How is supervision of CMAM activities carried out in the state?
- How many facilities do you visit each month?
- Are there other people involved in supervision of CMAM activities at state level?
    - If yes, what are their roles?
- What support is provided to you for supervision of CMAM activities at state level?
- What challenges do you face in supervision of CMAM activities at state level?

S1.4 Your training

- What is your level of education?
- When were you last trained in CMAM?
- What training were you given?
- Is there anything you were trained to do that is impractical, if so why?
- Does the position of State nutrition officer *(or UNICEF consultant, or M & E officer)* require you to do anything that you were not trained for?
- What changes would strengthen the training, to help you do your job well?

**S1.5 Supervision of your work**	
	• Who supervises your work for CMAM?<br>• How is the supervision organised?<br>• When were you last supervised?<br>• What is your opinion about the supervision?<br>• Is there anything you need to do for which you do not have sufficient guidance or supervision to do well?<br>• What changes in support would you suggest to meet your supervision and guidance needs?
**S1.6 Resources**	
	• Do you have enough time to achieve high quality service delivery?<br>*Probe for which CMAM or other tasks would benefit if the respondent had more time available.*<br>• Has the introduction of the SMS system for CMAM changed your workload? If yes how?<br>• Do your colleagues and management think it's important to have good systems to collect CMAM data and transfer CMAM data from the facility to higher levels?<br>• Do your colleagues and management value the use of information?<br>• Do you follow written guidelines/protocols? If so, please can you show them to us?<br>*Probe for Nigeria CMAM guidelines, training material, USAID guidelines*<br>• What additional resources would help you do your job related to CMAM better?<br>*Probe lack of resources e.g. computers/phones*

TOOL S2 Checklist/ observations about storing CMAM data at state level

State: **Date of visit:**
Name of person/people observed: **Observer:**

Please write notes in the spaces below each question. If you need more space, continue on a separate sheet using the question numbers on the left.

DATA STORAGE

**S2.1**	Are the data from CMAM facilities in each LGA integrated into LGA-wide reports?		**YES / NO**
**S2.2**	Do you keep paper copies of the monthly reports submitted by the LGAs? *If yes, ask to see the system of storage, and (depending on the response to question 2.1) ask to see an individual facility report from the first week of July 2017, or the LGA-wide integrated report for July 2017.*		**YES/ NO**
	Comment on how easy it was to find the report, and the filing system used for storing the reports		
**S2.3**	Are the reports locked up? *If no, go to Q2.5*		**YES/ NO**
**IF YES**	**S2.4**	Who has access to the key?	
		Name and job title	
**S2.5**	For how long do you keep the reports? *Ask to see the oldest report, and note the date*		
	Month and year of oldest monthly report stored. Comment how easy it was to find it		
**S2.6**	Is there written guidance on the length of time you should keep the reports? *If yes, ask to see the guidance, and note the content below.* If no, have you had verbal guidance? *If yes, summarise the verbal guidance below.*		**YES/ NO**<br>**YES/NO /n.a.**
	Guidance		
**S2.7**	Is there written guidance on methods of storing the reports so they remain confidential? *If yes, ask to see the guidance, and note the content below.* If no, have you had verbal guidance? *If yes, summarise the verbal guidance below.*		**YES/ NO**<br>**YES/NO /n.a.**
	Guidance		
**S2.8**	How do you dispose of the reports?		
	Disposal method		

TOOL S3 Checklist / observations about CMAM data collection and transfer at state level

**State:** **Date of visit:**<br>**Name of person/people observed:** **Observer:**<br><br>*Please write notes in the spaces below each question. If you need more space, continue on a separate sheet using the question numbers on the left.*		
**PAPER SYSTEM**		
**S3.1**	How many facilities in this State are supposed to be reporting each month?	**No of facilities……………..**
**S3.2**	How do you obtain the paper reports from the facilities?	
	Method	
**S3.3**	Is there a deadline for the LGAs to submit the CMAM monthly reports from the facilities to the State level? If so, what is it?	**YES Deadline ………… days after end of month / NO**
**S3.4**	Do you record the date that you collect or receive the CMAM reports from each LGA? *If NO go to question* 4.1	**YES/ NO**
	Check the dates of receipts for July 2017, and complete the tally on the right. (The total number of facilities should be the same as for Q3.1)	**Before deadline ………………<br>After deadline ………..<br>TOTAL………………**
**SMS SYSTEM**		
**S3.5**	Is there a deadline for submission of the facility weekly report to Federal level by SMS? If so, what is it?	**YES Deadline ………… days after end of month / NO**
**S3.6**	When the facilities submit their weekly reports by SMS to federal level, do they also inform the State office?	**YES / NO**
	If yes, check the dates that reports were sent for July 2017.<br>*(The total should be 4 x the number of facilities noted in Q3.1)*	**Before deadline ………………<br>After deadline ………..<br>TOTAL………………**
**GENERAL**		
**S3.7**	What are the advantages or disadvantages of the new SMS system compared to the paper-based system? Challenges of each?	
	Pros and Cons ***Challenges***	
**S3.8**	Are there others involved in collation of CMAM data at State level? **YES/NO**<br>If yes, who are they, and what are their roles?	
	People and job titles	

TOOL S4 Checklist / observations about CMAM data analysis at state level

**State:** **Name of person/people observed:**	**Date of visit:** **Observer:**	
Please write notes in the spaces below each question. If you need more space, continue on a separate sheet using the question numbers on the left.		
**S4.1**	Is there a manual to describe how to enter and analyse the data?<br>*If yes, ask to see it. If no, ask why and note response*	**YES/ NO**
	Comment	
**S4.2**	Do you find the software to be user-friendly?<br>*Ask for demonstration of data entry*	**YES/NO**
	Comment	
**S4.3**	Do you use any manual methods to minimise data entry errors? If yes, what are these methods? *If NO prompt for double data entry on all or a sample of data to check for errors, and ask for demonstration*	**YES/ NO**
	Comment	
**S4.4**	Are there automatic checks to avoid the wrong values for data being entered?<br>*IF NO prompt for data entry checks and logical error checks.*<br>*Ask for demonstration*	**YES/ NO**
	Comment	
**S4.5**	What procedures are used if data are missing?<br>*Ask for demonstration*	
	Comment	
**S4.6**	What procedures, if any, are used to clean the data?<br>*Ask for demonstration*	
	Comment	
**S4.7**	What procedures, if any, are used to check the quality of data?<br>*Ask for demonstration*	

	Comment	
**S4.8**	Do you record any data quality issues that you discover?<br>*If yes, ask to see how these are recorded*	**YES/NO**
	Comment	
**S4.9**	Does the database calculate any statistics automatically?<br>*If yes, ask for demonstration*	**YES/NO**
	Comment	
**S4.10**	Are the procedures for data cleaning, analysis and reporting documented in writing?<br>*If yes, ask to see the documentation*	**YES/NO**
	Comment	
**S4.11**	Are there systems for periodic data quality assessments?<br>*If yes, ask to see documentation*	**YES/NO**
	Comment	
**S4.12**	Have you changed how you analyse data, or how often, since the SMS system was introduced? Please explain	**YES/NO**
	Comment	
**S4.13**	Is there a continuous electricity supply?<br>If no, how often is the electricity supply interrupted?	**YES/NO Never/ Occasionally / Once a month / Twice a month / Weekly / Daily / n.a.**
**S4.14**	Is the room where the computer hardware is kept air-conditioned?<br>*Observe*	**YES/NO**
	Comment	

TOOL S5 Checklist / observations about data transfer from state to federal level

**State:** **Date of visit:**<br>**Name of person/people observed:** **Observer:**<br><br>*Please write notes in the spaces below each question. If you need more space, continue on a separate sheet using the question numbers on the left.*		
**S5.1**	After you aggregate LGA data, do you send the monthly compiled State reports to any individuals/ institutions by a certain deadline?<br><br>*If yes, record below who they are sent to, and what is the deadline*	**YES/NO**
	Reports sent	
**S5.2**	Is there a deadline for submission of the State monthly CMAM report to Federal level? IF YES what is it?	**YES Deadline = ………days after start of month /NO**
**S5.3**	Do you keep a record of transmitting monthly aggregated State reports to Federal level, with date?	**YES/NO**
	If yes, check the date of submission of the monthly State report to Federal level for January - June 2017, and complete tally on the right.	**Before deadline ………**<br>**After deadline …………**<br>**TOTAL 6……………….**
**S5.4**	How do you manage the transfer? Probe email attachment, dropbox	
	Method	

TOOL S6 Checklist about supervision and quality assurance of the CMAM programme at state level

**State:** **Date of visit:**<br>**Name of person/people observed:** **Observer:**<br>*Please write notes in the spaces below each question. If you need more space, continue on a separate sheet using the question numbers on the left.*		
**S6.1**	Do you keep records of your supervisory visits to facilities?<br>*If yes, check the records and note the dates of visits to facilities from LGA level during the last three months. Comment on form of notes kept. Then go to Q6.3*	**YES / NO**
	**Dates of visits** **Comment on record keeping**	
**S6.2**	If no, approximately how many supervisory visits to facilities from State level took place during the last three months?	**No visits / 1 / 2 / 3 / 4 or more**
**S6.3**	After visits, do you ever send feedback to facilities with advice or instructions?	**YES / NO**
	**Comment**	
**S6.4**	Are you informed if others make supervisory visits to facilities e.g. from LGA level?	**YES/ NO**
**S6.5**	What are the main challenges you face in your role with respect to quality control of CMAM data?<br>*Probe for capability, confidence, time, etc.*	
	Challenges	
**S6.6**	Compared to other tasks you have, how important is your role in quality control?	
	Priority	

TOOL S7 Checklist with about CMAM data use at state level

**State:** **Date of visit:**<br>**Name of person/people observed:** **Observer:**<br><br>*Please write notes in the spaces below each question. If you need more space, continue on a separate sheet using the question numbers on the left.*		
**S7.1**	Does the State office issue any report(s) containing CMAM data/ information?<br>*If yes, record details below of each report*	**YES/NO**
	Report title ***Frequency/ year*** ***Number of reports issues in past year***	
**S7.2**	• Does the State health office hold routine meetings to review the CMAM programme?<br>• Does the State health office hold routine meetings to review general managerial or administrative matters?<br><br>*Ask the following questions about the CMAM meeting, or if there is no CMAM meeting, ask about the general one:*<br>• Are you invited to attend the meeting?<br>• Do the nutrition officers from LGA level attend the meeting?<br>• How frequently is the meeting supposed to take place?<br>• How many times did the meeting take place during the last three months?<br>• Are official records of management meetings kept?<br>*If yes, check the record for the last meeting and note below which, if any CMAM-related issues were discussed*	**YES/NO**<br><br>**YES/NO**<br><br>**YES/ NO**<br>**Weekly/ Fortnightly / monthly/ quarterly /no schedule / n.a.**<br>**7 – 12 times / 3 - 6 times / once or twice / never / n.a.**<br>**YES/NO**
	Date of meeting and issues discussed	
**S7.3**	Did the State annual action plan for 2017 show decisions based on CMAM information? *If yes, note decisions below*	**YES/NO**
	Decisions	
**S7.3**	Please describe examples of how CMAM data are used at State level<br>*(if no examples are given, record NO EXAMPLES GIVEN, but then prompt to check if the CMAM data are used to forecast needs for training, supervision, supplies including RUTF)*	
	Details of data use	
**S7.6**	Would you like to use the CMAM data more, and if so, what are the challenges you face in doing so?	
	Challenges *(probe capability, confidence, time, lack of access)*	
**S7.7**	Are findings from analysis of CMAM data done at Federal level shared with you?<br>If YES, what findings are provided to you and how often?	**YES/NO**

	Description	
**S7.8**	If NO to Q7.7, would it be useful for you to be provided with findings from analysis of CMAM data done at Federal level? **If NO go to S7.14**	**YES/NO**
	Comments	
**S7.9**	Would you like to have findings from analysis of the CMAM data from the complete programme, or only the findings for this state?	**Complete programme/ Only this state**
	Comments	
**S7.10**	Which indicators would be most useful?<br>• Total in programme<br>• New admissions over a defined time period<br>• Cure rates<br>• As many indicators as possible	 **YES/NO**<br>**YES/NO**<br>**YES/NO**<br>**YES/NO**
	Comments	
**S7.11**	Would actual numbers, or summary graphs showing the trends be useful?<br>*Circle response on the right, and add any comments below*	**Exact numbers / Graphs / Both**
	Comments	
**S7.12**	How often should the reports be produced and provided to you? *Circle response on the right, and add any comments below*	**Weekly/ Monthly/ 3 mo./ 6 mo./ Year**
	Comments	
**S7.13**	Via which means would be best for you to get access to the reports? *Circle response on the right, and add any comments below*	**Email / Paper copy / Webpage**
	Comments	
**S7.14**	Are there other potential users of the data/ information that currently do not have access?<br>If yes, who are they (level) and what would they use the data for?	**YES/NO**
	Individuals or institutions ***Potential use***	
**S7.15**	Does the State office display any CMAM data? *If yes, indicate types of data displayed below, and if data are updated for last reporting period on the right*<br>Table **YES/NO** Data type:…………………………………………. Updated? **YES/NO**<br>Graph/Chart **YES/NO** Data type:…………………………………………. Updated? **YES/NO**<br>Map **YES/NO** Data type:……………………………………………. Updated? **YES/NO**	**YES/NO**

Tool S8 Interviews with potential users of data at State level

**Location:** **Date of interview:** **Interviewer:**<br>**Name of interviewee:** **Job title:**<br><br>*Please write notes in the spaces below each question. If you need more space, continue on a separate sheet using the question numbers on the left.*		
**S8.1**	Would you be interested to receive reports of information derived from CMAM programme data collected from facilities in this LGA / State?<br><br>*(Only for users at State level)* Would you be interested to receive reports of information derived from CMAM programme data collected from all the NE and NW states?	**YES/NO**<br><br>**YES/NO / n.a.**
**S8.2**	If yes, which indicators would be most useful?<br>• Total in programme<br>• New admissions over a defined time period<br>• As many indicators as possible	 **YES/NO**<br>**YES/NO**<br>**YES/NO**
	Comments	
**S8.3**	Would it be useful if the indicators were reported by groups, for example by LGA or State? *Add any comments below*	**YES/ NO**
	Comments	
**S8.4**	Would actual numbers, or summary graphs showing the trends be useful? *Circle response on the right, and add any comments below*	**Exact numbers / Graphs /Both**
	Comments	
**S8.5**	How often should the reports be produced and provided to users? *Circle response on the right, and add any comments below*	**Weekly/ Monthly/ Every 3 mo. / 6 months/ Year**
	Comments	
**S8.6**	Which method would be best for you to get the reports? *Circle response on the right, and add any comments below*	**Email / Paper / Webpage**
	Comments	
**S8.7**	What would you use the information for?	
	Potential use of information	
**S8.8**	***Any other comments***	

Location: **Date of interview:** **Interviewer:**
Name of interviewee: **Job title:**

Question number	

FEDERAL LEVEL

TOOL N1 Interview with MoH / UNICEF information officer

Please write notes on the responses using separate sheets, using the question numbers on the left.	
**N1.1 Your role**	
	• How long have you been in this post? Can you please explain your role?<br>• Approximately what proportion of your time is spent on the CMAM programme?
**N1.2 CMAM information system operation**	
	• How many facilities offer CMAM services?<br>• How many facilities send data to Federal level using the new SMS system?
**N1.3 CMAM Supervision of facilities**	
	• Please explain the system for keeping updated at Federal level on activities regarding supervision at facilities.
**N1.4 CMAM Supervision of LGAs**	
	• Please explain the system for keeping updated at Federal level on activities regarding supervision at LGA level.
**N1.5 Your training**	
	• What is your level of education?<br>• Have you attended any training in CMAM? If yes, what training?<br>• Have you attended (or led) any training in the CMAM information system? If yes, specify.<br>• Does the position of …………………………………… require you to do anything for which you have not been taught the necessary skills?<br>• What training would you like to receive, to help you do your job well?
**N1.6 Supervision of your work**	
	• Who supervises your work for the CMAM information system?<br>• How is the supervision organised?<br>• What is your opinion about the supervision?

	• Is there anything you need to do for which you do not have sufficient guidance or supervision to do well?<br><br>• What changes in support would you recommend to meet your supervision and guidance needs?
**N1.7 Resources**	
	• Do you have enough time to achieve high quality service delivery? *Probe for which CMAM or other tasks would benefit if the respondent had more time available.*<br><br>• Has the introduction of the SMS system for CMAM changed your workload? If yes how?<br><br>• Do your colleagues and management think it's important to have good systems to collect CMAM data and transfer CMAM data from the facility to higher levels?<br><br>• Do your colleagues and management value the use of information?<br><br>• Do you follow written guidelines/protocols? If so which ones?<br>*Probe for Nigeria CMAM guidelines, training material, USAID guidelines*<br><br>• What additional resources would help you do your job related to CMAM better?<br><br>• Is there a lack of resources at any level that hinders smooth running of the CMAM information system? *If yes, specify level, and resource e.g. Computers/ Phones / Staff*
**N1.8 SMS system**	
	• In your view what is the greatest advantage, and the greatest disadvantage, of the SMS system compared to the paper-based system?<br><br>• What are the main challenges the MoH faces taking over the information system?

TOOL N2 Checklist / observations about CMAM data transfer to federal level

Location: **Date of visit:**
Name of person/people observed: **Observer:**

Please write notes in the spaces below each question. If you need more space, continue on a separate sheet using the question numbers on the left.

PAPER SYSTEM

**N2.1**	Does the Ministry of Health keep electronic copies of the CMAM monthly reports sent from state-level?<br>*If yes look at reports from July 2017*	**YES/ NO**
	Comments on storage system and ease of retrieval	
**N2.1**	How many States are supposed to be reporting each month?	**No of states……………..**
**N2.2**	How many states actually did send in reports for July 2017?	**No of states………………**
**N2.3**	Is there a deadline for submission of the CMAM electronic monthly report by state? If yes, what is it? *Note deadline on the right.*	**YES Deadline ………… days after end of month / NO**
**N2.4**	Do you record the date that you received the CMAM electronic monthly reports from each state? *If NO go to question* 2.5	**YES/ NO**
	Check the dates of receipts for July 2017, and complete the tally on the right. (The total number of States should be the same as for Q2.1)	**Before deadline ……………….**<br>**After deadline …………**<br>**TOTAL……………….**

SMS SYSTEM

**N2.5**	Is there a deadline for submission of the facility weekly report to Federal level by SMS? If yes, what is it? *Note deadline on the right.*	**YES Deadline ………… days after end of week / NO**
	If yes, check the dates that reports were sent for the first week of July. (Can complete this later from the database)	**Before deadline …………….**<br>**After deadline …………**<br>**TOTAL…………….**

GENERAL

**N2.7**	What are the advantages or disadvantages of the new SMS system compared to the paper-based system? Challenges of each? *This can include data analysis, not just data transfer*
	Pros and Cons ***Challenges***

TOOL N3 Checklist / observations about CMAM data analysis at federal level

**Location:** **Name of person/people observed:**	**Date of visit:** **Observer:**	
Please write notes in the spaces below each question. If you need more space, continue on a separate sheet using the question numbers on the left.		
**PAPER SYSTEM**		
**N3.1**	Is there a manual to describe how to enter and analyse the data? *If yes, ask to see it*	**YES/ NO**
	Comment	
**N3.2**	Do you find the software to be user-friendly? *Ask for demonstration of data entry*	**YES/NO**
	Comment	
**N3.3**	Do you use any methods to check for data errors/ data quality? If yes, what are these methods? A*sk for demonstration.* *If answer is no, prompt for logical checks, plotting data*	**YES/ NO**
	Comment	
**N3.5**	What procedures are used if data are missing? *Ask for demonstration*	
	Comment	
**N3.6**	What procedures, if any, are used to clean the data? *Ask for demonstration*	
	Comment	
**N3.7**	What procedures, if any, are used to check the quality of data? *Ask for demonstration*	
	Comment	
**N3.8**	Do you record any data quality issues that you discover? *If yes, ask to see how these are recorded*	**YES/NO**
	Comment	
**N3.9**	Does the database calculate any statistics automatically? *If yes, ask for demonstration*	**YES/NO**

	Comment	
**N3.10**	Are the procedures for data cleaning, analysis and reporting documented in writing? *f yes, ask to see the documentation*	**YES/NO**
	Comment	
**N3.11**	Are there systems for periodic data quality assessments? *If yes, ask to see documentation*	**YES/NO**
	Comment	
**N3.12**	Have you changed how you analyse data, or how often, since the SMS system was introduced? Please explain	**YES/NO**
	Comment	
**SMS SYSTEM**		
**N3.13**	Is there a manual to describe how to enter and analyse the data? *If yes, ask to see it*	**YES/ NO**
	Comment	
**N3.14**	Do you find the software to be user-friendly? *Ask for demonstration of data entry*	**YES/NO**
	Comment	
**N3.15**	Do you use any methods to check for data errors/ data quality? If yes, what are these methods? If yes, what are these methods? A*sk for demonstration. If answer is no, prompt for logical checks, plotting data*	**YES/ NO**
	Comment	
**N3.16**	What procedures are used if data are missing? *Ask for demonstration*	
	Comment	
**N3.17**	What procedures, if any, are used to clean the data? *Ask for demonstration*	
	Comment	
**N3.18**	What procedures, if any, are used to check the quality of data? *Ask for demonstration*	
	Comment	

**N3.19**	Do you record any data quality issues that you discover?<br>*If yes, ask to see how these are recorded*	**YES/NO**
	Comment	
**N3.20**	Does the database calculate any statistics automatically?<br>*If yes, ask for demonstration*	**YES/NO**
	Comment	
**N3.21**	Are the procedures for data cleaning, analysis and reporting documented in writing? *f yes, ask to see the documentation*	**YES/NO**
	Comment	
**N3.22**	Are there systems for periodic data quality assessments?<br>*If yes, ask to see documentation*	**YES/NO**
	Comment	
**N3.23**	What system do you use to keep the data secure?	
	Comment	

TOOL N4 Checklist about quality assurance of the CMAM programme at federal level

**Location:** **Date of visit:**<br>**Name of person/people observed:** **Observer:**<br>*Please write notes in the spaces below each question. If you need more space, continue on a separate sheet using the question numbers on the left.*		
**N4.1**	Do you provide feedback to state level with respect to the quality of the data (paper and /or SMS systems) sent to you?<br>*If yes, ask to see examples of report or message.*	**YES / NO**
	**Comment**	
**N4.2**	Do you have meetings with State nutrition officers at which you discuss data issues? *If yes, ask to see minutes from the last meeting*	**YES / NO**
	**Comment**	
**N4.3**	What are the main challenges you face in your role with respect to quality control of CMAM data?<br>*Probe capability, confidence, time.*	
	Challenges	
**N4.4**	Compared to other tasks you have, how important is your role in quality control?	
	Priority	

TOOL N5 Checklist with about data use at federal level

**Location:** **Date of visit:**<br>**Name of person/people observed:** **Observer:**<br><br>*Please write notes in the spaces below each question. If you need more space, continue on a separate sheet using the question numbers on the left.*		
**N5.1**	Do you summarise findings from the CMAM programme data analysis and provide them to nutrition stakeholders? *If No, go to Q5.3*<br>*If yes, note the reports below and ask how for demonstration of the reports are prepared.*	**YES/NO**
	Report title ***Frequency/ year*** ***Number of reports issues in past year***	
**N5.2**	If YES to Q5.1, to whom do you provide these findings?	
	Individuals or institutions	
**N5.3**	Does the Ministry of Health / UNICEF hold routine meetings to review the CMAM programme? *Ask the following questions about the CMAM meeting, or if there is no CMAM meeting, ask about the general one:*<br>• Are you invited to attend the meeting?<br>• *(for MoH)* Do the State nutrition officers attend the meeting?<br>• How frequently is the meeting supposed to take place?<br>• How many times did the meeting take place during the last three months?<br>• Are official records of management meetings kept?<br>*If yes, check the record for the last meeting and note below which, if any CMAM-related issues were discussed*	**YES/NO**<br><br>**YES/NO**<br><br>**YES/ NO**<br>**Weekly/ Fortnightly / monthly/ quarterly /no schedule / n.a.**<br>**7–12 times / 3- 6 times/ once or twice /never/ n.a.**<br>**YES/NO**
	Date of meeting and issues discussed	
**N5.4**	Did the Federal annual health plan for 2017 show decisions based on CMAM information? *If yes, note decisions below*	**YES/NO**
	Decisions	
**N5.5**	Please describe examples of how the Ministry of Health / UNICEF uses CMAM data	
	Details of data use	
**N5.6**	Would you like to use the CMAM data more?<br>If yes, what are the challenges you face in doing so?	**YES/NO**
	Challenges *(probe capability, confidence, time)*	
**N5.7**	Are there other potential users of the data/ information that currently do not have access? If yes, who are they (level) and what would they use the data for?	**YES/NO**
	Individuals or institutions ***Potential use***	

Tool N6 Interviews with potential users of data at federal level e.g. WFP, NGOs

Location: **Date of interview:** **Interviewer:**
Name of interviewee: **Job title:**
Please write notes in the spaces below each question. If you need more space, continue on a separate sheet using the question numbers on the left.

**F6.1**	Do you have direct access to the summary reports on the dashboard, derived from the CMAM programme data? **If No, go to Question F6.6**			**YES/NO**
IF YES to F6.1	**F6.2**	Do you use the information from the dashboard reports?<br>If yes, please explain what for. **If No, go to Question F6.6**		**YES/NO**
		Current use of information		
	IF NO to F6.2	**F6.3**	What are the main reasons why you don't use the information from the dashboard reports?<br>• I have difficulties in analysing the data because of lack of training/skills<br>• I have difficulty in analysing the data because of lack of technology<br>• I have difficulty in interpreting the findings<br>• I have other work priorities<br>• I don't trust the data<br>• Other (please describe below)	**YES/NO**<br>**YES/NO**<br>**YES/NO**<br>**YES/NO**<br>**YES/NO**
			Comments	
	**F6.4**	Are the data updated often enough to inform program management decisions?		**YES/ NO**
		Comments		
	**F6.5**	Is there enough detail? If not, what additional information would be helpful?<br>**Now go to Question F6.8**		**YES/ NO**
		Additional information		
IF NO to F6.1	**F6.6**	Would you like to have direct access to the summary reports on the dashboard?<br>**If No, go to Question F6.8**		**YES/ NO**
	**F6.7**	Would you use the information from the reports?		
		Potential use of information		
**F6.8**	Would it be useful to you / your organization if summary reports derived from the CMAM monitoring data were regularly provided to you (rather than accessing the dashboard yourself)? **If No, go to Question F6.15**			**YES/ NO**
	Comments			
**F6.9**	Which indicators would be most useful?<br>• Total in programme<br>• New admissions over a defined time period			**YES/NO**<br>**YES/NO**<br>**YES/NO**

	• As many indicators as possible	
	Comments	
**F6.10**	Would it be useful if the indicators were disaggregated, for example by LGA /State?	**YES/ NO**
	Comments	
**F6.11**	Would actual numbers, or summary graphs showing the trends be useful?<br>*Circle response on the right, and add any comments below*	**Exact numbers<br>/ Graphs<br>/ Both**
	Comments	
**F6.12**	How often should the reports be produced and provided to users?<br>*Circle response on the right, and add any comments below*	**Weekly/<br>Monthly/<br>3 mo. / 6 months/ Year**
	Comments	
**F6.13**	Via which means would be best for you to get access to the reports?<br>*Circle response on the right, and add any comments below*	**Email<br>/ Paper copy<br>/ Webpage**
	Comments	
**F6.14**	What would you use the information for?	
	Potential use of information	
**F6.15**	***Any other comments***	

**Location:**	**Date of interview:**	**Interviewer:**
**Name of interviewee:**	**Job title:**	

Question number	

Tool F10a: Protocol for verification of CMAM admission and discharge data

Summary of the task

To obtain a better understanding of the accuracy of the CMAM reporting on admissions to OTPs, children in treatment and children discharged, we are going to recount and tally a small sample of the CMAM records in a selection of facilities, with the aim of comparing our counts to the data found in CMAM monthly (for paper-based system) and weekly (for SMS-system) databases. The verification will mostly be done on the basis of past cases, so that it can be undertaken while CMAM treatment of current cases and admissions of new cases are happening at the facility.

We will:

A) Recount admissions

- Count cards with admissions between **April 2017 and June 2017** to get an overall count by of cards by admission month to compare with the CMAM registration book (if available) AND/OR to compare with data in CMAM facility-level data sets.
    - ***Why are we doing this?*** We want to assess whether OTP cards in facilities disappear.

- Count children disaggregated by specific age and admission groups and admitted in **July 2017** based on the admission date on the front of the CMAM OTP cards.
    - ***Why are we doing this?*** We want to obtain a complete recount of cases admitted in July with the same disaggregations as presented in the CMAM records from SMS and paper, to assess any discrepancies.

B) Capture information about re-admitted defaulters and relapses

- For children admitted in **July 2017**, generate a count of cases registered on OTP cards as re-admitted defaulters or newly admitted as relapses
    - ***Why are we doing this?*** The current CMAM data system does not feed information about re-admitted defaulters and relapses up to LGA/state/federal level. We want to understand how often these cases are reported.

C) Assess consistency in reporting on dates on OTP cards and treatment characteristics

- For admissions in **July 2017**, check **consistency** in terms of admission dates - Count children admitted in a given month based on admission date on the **back of the CMAM OTP cards;**
    - ***Why are we doing this?*** Dates are recorded twice, which is a possible source for error.
- *Lower priority – do if time allows:* For admissions in July 2017, check sequence of weeks in treatment and recording of absences
    - ***Why are we doing this?*** Assess whether treatment dates are consistent (i.e. every 7 days); and if absences are recorded correctly.

D) Discharges and treatment duration based on OTP cards

- Count children on OTP cards who **exited** in **July 2017**, differentiating whether they were cured, defaulted, died, recorded as non-recovered, transferred, or discharge reason is unknown;
    - ***Why are we doing this?*** We want to obtain a complete recount of cases discharged in July with the same disaggregations as presented in the CMAM records from SMS and paper, to assess any discrepancies.
- For recovered cases, note down number of weeks in treatment
    - ***Why are we doing this?*** UNICEF/CIFF brought up anecdotal evidence that children are discharged too quickly and would like to explore this issue.

E) Scan forms

- **Scan weekly and monthly tallies** prepared by the CMAM staff at the facility for **April, May, June and July 2017** for later comparison with the aggregate monthly or weekly data in databases;
- If available, scan records for **April, May, June and July 2017** from **CMAM registration book**
- Scan **selected OTP admission cards** to highlight errors

F) RUTF

- In a subset of selected facilities, assess the various RUTF receipt and issuance records in RUTF stock cards, OTP cards, and ration cards.
    - ***Why are we doing this?*** UNICEF/CIFF are concerned about RUTF misreporting and leakage

The data verification task is easier if two people work together, a CALLER and a TALLYER.

- The CALLER is responsible for looking through the CMAM OTP cards and calls out relevant entries
- The TALLYER is responsible for marking tallies in the CMAM verification data collection forms.

Preparatory steps

1. Examine card storage system and note how cards are sorted – they will have to be put back in the same way, and we will have to understand the sorting mechanism in order to identify the correct set of cards. Based on experience from visits to other CMAM centres, the most common sorting structure is by the date of admission/re-admission.

    - Past cases should have folders for cured, died, defaulted, non-recovered; establish whether these are sorted by day of exit or day of admission. This might be different in practice.

    - If sorted by day of first admission, take all cards covering for first admissions/admission after relapse in **April, May, June, and July**, according to the admission date on the front of the OTP card. This will enable us to do a recount of admissions for April, May, June and July, and exits for the month of July.

You will need a desk or other flat space where you can lay out cards.

Tallying CMAM OTP admissions from CMAM OTP cards

2. CALLER:
    - Sort all cards with the front-page up and **check the date of admission**. Create one pile of cards for each month (April, May, June, July), i.e. four piles in total.
    - Pass on any cards with admission dates that are NOT in April, May, June or July to TALLYER.

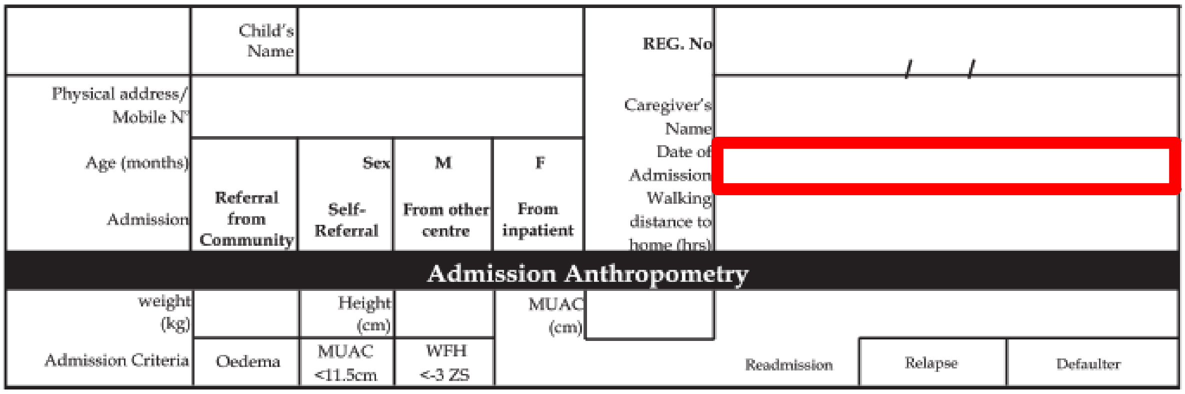
Child's Name
REG. No
/ /
Physical address/ Mobile N°
Caregiver's Name
Age (months)
Sex
M
F
Date of Admission
Admission
Referral from Community
Self-Referral
From other centre
From inpatient
Walking distance to home (hrs)
Admission Anthropometry
weight (kg)
Height (cm)
MUAC (cm)
Admission Criteria
Oedema
MUAC <11.5cm
WFH <-3 ZS
Readmission
Relapse
Defaulter

TALLYER:
- Double-check cards passed by CALLER and ensure no admissions fall within the four months, then discard on a separate pile.

OPTION 1 – COUNTING CARDS FOR APRIL, MAY, JUNE: ***FAST VERSION: if little time is available obtain total count by month, for comparison with records from paper.***

TALLYER:
- From the list at the back of this document, record relevant calendar weeks covered in the given month (accounting for CMAM open day) on top of "**SECTION 0: COUNTING ADMISSIONS FROM OTP CARD FOR APRIL, MAY, JUNE**".

CALLER:
- Count cards on each pile for April, May and June and state number to TALLYER.
- Hand the three piles of cards to TALLYER.

TALLYER
- Note down number on separate piece of paper
- Re-count number of cards on each pile for April, May, and June.
- If the two counts match, record total on top of "**SECTION 0: COUNTING ADMISSIONS FROM OTP CARD FOR APRIL, MAY, JUNE**", under **Option 1**. If not, recount.

COUNTING ADMISSIONS FROM OTP CARD (FRONT) FOR 3 MONTHS			
	**Apr-2017**	**May-2017**	**Jun-2017**
	**Calendar weeks:**	**Calendar weeks:**	**Calendar weeks:**
**Total (Option 1)**	TOTAL:	TOTAL:	TOTAL:

OPTION 2 – COUNTING CARDS FOR APRIL, MAY, JUNE: ***If you assess that sufficient time is available, perform weekly count that can also be compared with weekly SMS records***

TALLYER:

- Enter week start/end dates in the “**SECTION 0: COUNTING ADMISSIONS FROM OTP CARD FOR APRIL, MAY, JUNE”**, under **Option 2**.

CALLER:

- For each card, call out admission date

TALLYER:

- Check which week the date falls in
- Tally each date in the correct week box in the **SECTION 0** sheet “**SECTION 0: COUNTING ADMISSIONS FROM OTP CARD FOR APRIL, MAY, JUNE**”, under Option 2.

Week/start end:							Week/start end:							Week/start end:						
𝍸	III																			
					TOTAL:							TOTAL:							TOTAL:	

3. TALLYING DETAILS OF ADMISSIONS FOR JULY

CALLER: Sort all cards with admission date in July with the front-page up and **check age at admission and admission type**; sort cards into the following batches:

- Admissions aged 6-59 in batch 1 (referral from community, self-referral)
- From outpatient care aged 6-59 in batch 2 (from other OTP centre)
- From inpatient care aged 6-59 in batch 3 (from inpatient)
- Admissions other ages in batch 4 (referral from community, self-referral)
- From outpatient care other ages in batch 5 (from other centre)
- From inpatient care other ages in batch 6 (from inpatient)

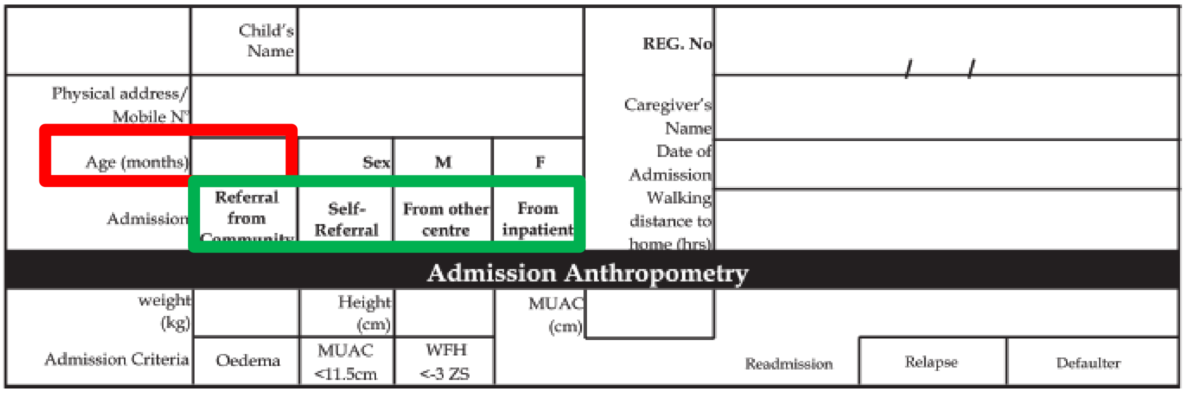

	Child's Name				REG. No	/ /	
Physical address/ Mobile N°					Caregiver's Name		
Age (months)		Sex	M	F	Date of Admission		
Admission	Referral from Community	Self-Referral	From other centre	From inpatient	Walking distance to home (hrs)		
Admission Anthropometry							
weight (kg)		Height (cm)		MUAC (cm)			
Admission Criteria	Oedema	MUAC <11.5cm	WFH <-3 ZS		Readmission	Relapse	Defaulter

While the new system based on SMS limits reports to children aged 6-59 months, we still collect information about other age groups. This will allow us to check whether staff are correctly excluding children of other age groups from the reporting. For the same reason we also collect more detailed information on the admission type (distinguishing transfers from OTPs and inpatient care).

CALLER:

- If there are cards left that have **no circle** in any of the admission criteria (referral from community, self-referral, from other centre, from inpatient), check if either “relapse” or “defaulter” are circled.
  - If yes, sort cards into up to four additional piles:
    - Relapse 6-59 months
    - Relapse Other ages
    - Defaulter 6-59 months
    - Defaulter Other ages
  - If no, sort cards into up to two additional piles:
    - Unknown, 6-59 months
    - Unknown, other ages
- If there are cards with relapse or defaulter is circled AS WELL as another category: classify them into one of the six major categories described initially.
- 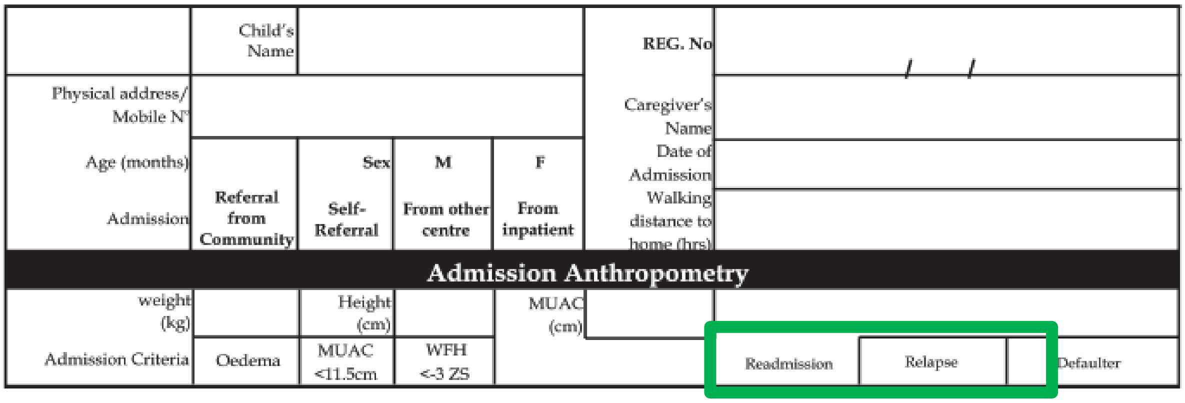

	Child's Name				REG. No	/ /	
Physical address/ Mobile N°					Caregiver's Name		
Age (months)		Sex	M	F	Date of Admission		
Admission	Referral from Community	Self-Referral	From other centre	From inpatient	Walking distance to home (hrs)		
Admission Anthropometry							
weight (kg)		Height (cm)		MUAC (cm)			
Admission Criteria	Oedema	MUAC <11.5cm	WFH <-3 ZS		Readmission	Relapse	Defaulter

Calling and recording of admissions

4. CALLER:
   - Start with batch of new admissions aged 6-59 months; call out type of admission to TALLYER
   - For each card, call out admission date

5. TALLYER:
    - Check which week the date falls in
    - Tally in the correct week box in the CMAM OTP data verification collection form – SECTION 1 (**TALLYING ADMISSIONS FOR JULY 2017 FROM OTP CARD**); make sure you always tally in groups of 5.
    - If the caller is calling too quickly, ask them to slow down.

	**Insert Calendar Week number…**
New admissions 6-59 months	INSERT start/end of week, e.g. 03/07 – 09/07
	~~IIII~~ III
	1
	**TOTAL:**

6. CALLER:
    - To verify consistency of dates, turn card around and call out date under ADM (1)

FOLLOW UP: OUTPATIENT THERAPEUTICAL PROGRAMME

Name						
Visits	ADM (1)	2	3	4	5	6
Date						

7. TALLYER:
    - Notice if date differs from previous date; if yes say “inconsistent date”
    - Tally inconsistencies in the boxes at the bottom of the sheet

INCON-SISTENT DATE ON BACK	
	**TOTAL:**

    - If you are uncertain, ask CALLER to repeat both front and back dates.

8. CALLER:
    - Turn card back to front.
    - For admission types referral from community, self-referral, from other centre, from inpatient, check if either the relapse or the defaulter box are **ALSO** ticked (these are treated differently from cards which had ONLY relapse or default ticked).
    - After having called out the date, call out “relapse” / “defaulter”

9. TALLYER:
    - Tally additional ticks on defaulters or relapses in the following boxes

Defaulter also ticked		**TOTAL:**		**TOTAL:**		**TOTAL:**
Relapse also ticked		**TOTAL:**		**TOTAL:**		**TOTAL:**

10. Repeat the same process (calling dates and tallying by week) for the card batches for other admission types.
    - Admissions for ages 6-59 months are on SECTION 1-1
    - Admissions for other ages are on SECTION 1-2

11. If you have verified all cards:
    - TALLYER adds totals for weeks in the total boxes.
    - Go to next section of protocol (“SECTION 2: CHECKING SEQUENCE OF TREATMENT DATES AND ABSENCES”) – if time allows, otherwise go to SECTION 3

Lower priority – do if time allows: For admissions in July 2017, check sequence of weeks in treatment and recording of absences

CALLER:

12. Turn all cards in pile for JULY admissions facing with the back side of the card up.
13. Check whether treatment days recorded are consecutive, i.e. take place **every 7 days. For this check dates on top and whether weight and MUAC are recorded.**
14. If visits are not consecutive or the column is empty (as for instance in the example below for 25/7); say non-consecutive

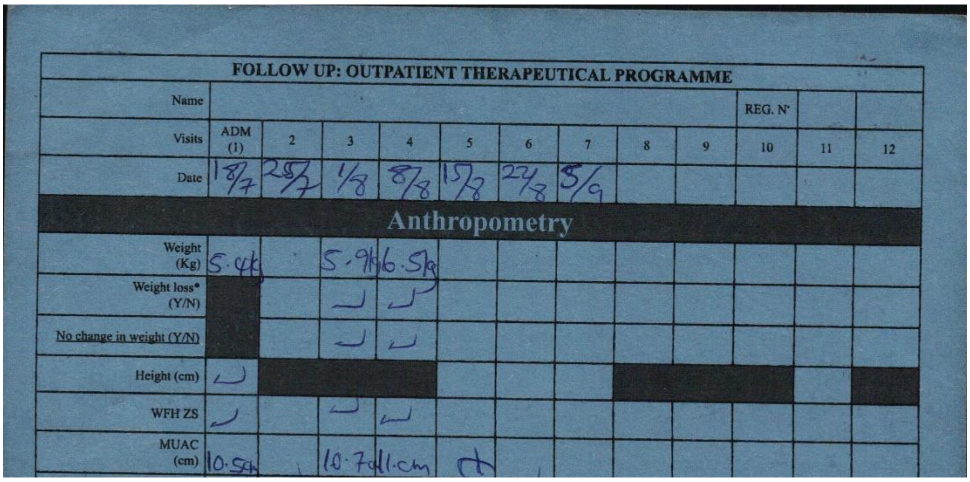

FOLLOW UP: OUTPATIENT THERAPEUTICAL PROGRAMME

Name									REG. N°			
Visits	ADM (1)	2	3	4	5	6	7	8	9	10	11	12
Date	18/7	25/7	1/8	8/8	15/8	22/8	5/9					
**Anthropometry**												
Weight (Kg)	5.4kg		5.9kg	6.5kg								
Weight loss* (Y/N)			✓	✓								
No change in weight (Y/N)			✓	✓								
Height (cm)	✓											
WFH ZS	✓		✓	✓								
MUAC (cm)	10.5cm		10.7cm	11.cm	[illegible]							

TALLYER:

15. Tally on “SECTION 2: CHECKING SEQUENCE OF TREATMENT DATES AND ABSENCES” under Non-consecutive visits

CALLER:

16. Now check whether absences were recorded for non-consecutive weeks (see example below, or should more correctly be in “Outcome”)

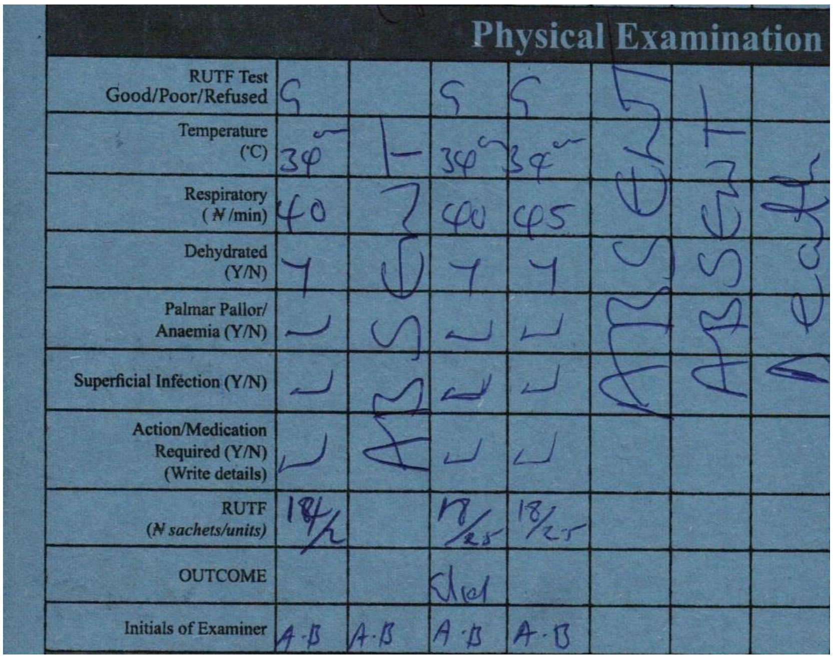

Physical Examination							
RUTF Test Good/Poor/Refused	G		G	G	ABSENT	ABSENT	[illegible]
Temperature (°C)	34	—	34	34			
Respiratory (N/min)	40		40	45			
Dehydrated (Y/N)	Y	ABSENT	Y	Y			
Palmar Pallor/ Anaemia (Y/N)	N		N	N			
Superficial Infection (Y/N)	N		N	N			
Action/Medication Required (Y/N) (Write details)	N		N	N			
RUTF (N sachets/units)	18/2		18/25	18/25			
OUTCOME			[illegible]				
Initials of Examiner	A.B	A.B	A.B	A.B			

17. If Absence is recorded, say “absent”

TALLYER:

18. Tally recorded absences on “SECTION 2: CHECKING SEQUENCE OF TREATMENT DATES AND ABSENCES” in the right-hand side column.
19. Once all cards have been checked, compute total.

SECTION 2: CHECKING SEQUENCE OF TREATMENT DATES AND ABSENCES														
	Non-consecutive visits							Absence recorded						
						TOTAL:							TOTAL:	

Tallying of discharges by type of discharge

CALLER:

20. Bring again all card piles (admissions in April, May, June, and July) in front of you.

21. Turn all cards facing with the back side of the card up.

CALLER:

22. Find the last date recorded on top of the card.

23. Check which month the date falls in. Hand card to TALLYER if month is NOT JULY 2017.

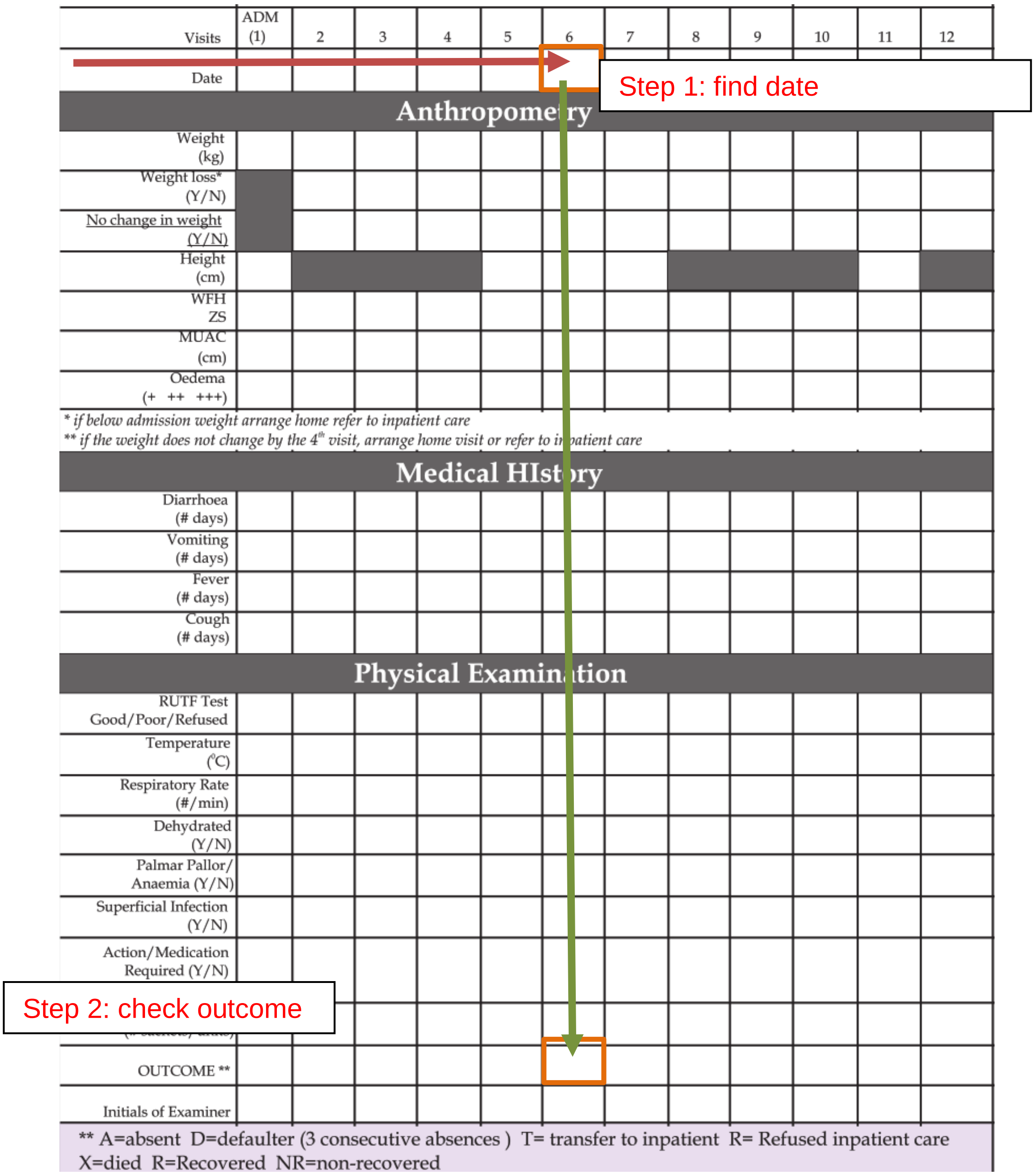

Visits	ADM (1)	2	3	4	5	6	7	8	9	10	11	12
Date												
**Anthropometry**												
Weight (kg)												
Weight loss* (Y/N)												
No change in weight (Y/N)												
Height (cm)												
WFH ZS												
MUAC (cm)												
Oedema (+ ++ +++)												
* *if below admission weight arrange home refer to inpatient care* ** *if the weight does not change by the 4th visit, arrange home visit or refer to inpatient care*												
**Medical HIstory**												
Diarrhoea (# days)												
Vomiting (# days)												
Fever (# days)												
Cough (# days)												
**Physical Examination**												
RUTF Test Good/Poor/Refused												
Temperature (°C)												
Respiratory Rate (#/min)												
Dehydrated (Y/N)												
Palmar Pallor/ Anaemia (Y/N)												
Superficial Infection (Y/N)												
Action/Medication Required (Y/N)												
[illegible]												
OUTCOME **												
Initials of Examiner												

** A=absent D=defaulter (3 consecutive absences) T= transfer to inpatient R= Refused inpatient care X=died R=Recovered NR=non-recovered

TALLYER:

24. Double-check month. If confirmed as falling outside July, discard card.

CALLER:

25. For cards with last dates in July, check code for outcome reason and separate into different batches.
    - R: Recovered ⟶ Batch 1
    - X: Died ⟶ Batch 2
    - D: Defaulter ⟶ Batch 3
    - NR: Non-recovered ⟶ Batch 4
    - T: Transfer to inpatient care ⟶ Batch 5

    If there is no entry in outcome row or elsewhere on the card, separate into up to three additional categories:
    - Reason unspecified and unclear - Batch 7

- Reason unspecified and likely recovered – Batch 8
- Not discharged but meet criteria for defaulting – Batch 9

Don't worry too much about correctly classifying children into these last three categories since the categories will be aggregated for the verification analysis.

The purpose of separating them is to enable estimates of recovery and defaulting rates.

26. Now go through batch by batch and call out date.

TALLYER:

27. Check which week the date falls in.
28. Tally in the correct week box in the **CMAM OTP data verification collection form – SECTION 3 (TALLYING DISCHARGES)**; make sure you always tally in groups of 5.
29. If the caller is calling too quickly, ask them to slow down.

30. Repeat the same process (calling dates and tallying by week) for the card batches for other discharge types (Batches 2, 3, 4, 5, 6).
31. TALLYER adds totals for weeks in the total box.

32. **Only for the recovered children** (Batch 1) note the number of weeks in treatment.

MAKE SURE THAT YOU FILL IN THE COVER SHEET!

Take scans of documents for later verification and entry

In order to assess whether weekly tallies were correctly aggregated to monthly site summaries, and monthly site summaries were correctly reflected in the facility-level databases, we capture weekly tallies and monthly site summaries. We also capture CMAM registration books to compare counts against tallies done in SECTION 1.

- Ask to see:
    - weekly tally sheets for the months of April, May, June and July 2017
    - monthly site summaries for the months of April, May, June and July 2017
    - CMAM registration book if available
- Scan weekly tally sheets and monthly site summaries for April, May, June and July 2017;
- Find pages in registration book for admissions in April, May, June and July 2017 and photograph.

Verification of RUTF distribution

Consider time requirements. The RUTF verification can take place in a subset of facilities.

Verification of stocks and flows on the day of visit - Tool F10c Sections 1, 2 and 3

If you have a laptop some records can be captured directly in the excel form rather than on paper.

SECTION 1 – COUNT FROM STOCKS AND TALLIES AT THE BEGINNING AND END OF THE DAY:

Only do steps 1-4 if you arrive before the first caregivers and stay until the end of the CMAM treatment day.

1. Count boxes and sachets of RUTF stock at the beginning of the OTP day. To minimize errors, have a second person repeat the count.
2. Record morning count.
3. Count boxes and sachets of RUTF stock at the end of the OTP day. To minimize errors, have a second person repeat the count.
4. Record evening count.
5. Ask to see RUTF stock card entries for previous week and current week, as well as and weekly tally sheet for current week (after OTP team has finalized tallies); scan forms.
6. Capture balance of RUTF at the end of the previous week from RUTF Stock card.
7. Capture RUTF utilised based on RUTF stock cards and weekly tallies.

SECTION 2 – COUNT FROM OTP CARDS - AT THE END OF THE DAY:

1. Ask to see OTP cards used during the day.
2. Transfer number of sachets given to children from back of the card to SECTION 2.

RUTF (# sachets/units)						
OUTCOME **						
Initials of Examiner						

SECTION 2: RECOUNT RUTF USE ON DAY OF VISIT FROM OTP CARDS										
	Sachets disbursed on day									
	11	14	12							

SECTION 3: COMPARISON OF RUTF RECEIVED, RUTF RATION CARDS AND OTP CARDS – DURING THE DAY

For a limited number of caregivers (up to 8 per facility):

1. Note down number of sachets allocated according to OTP card (observation)
2. Once caregiver has received the RUTF and is leaving the facility
    a. Ask to see the RUTF received for a specific child and count and note down number of sachets
    b. Ask to see the caregiver's RUTF ration card and note down RUTF allocated
3. Record the three entries in SECTION 3.

Tallying RUTF from OTP cards for month of July – following admission/discharge verification; Tool F10c Section 4

This task requires a considerable amount of time, consider whether feasible.
You work again together as CALLER and TALLYER.

CALLER:

1. Have cards with admission in April, May, June and July turned around so that back of the card is facing up
2. Go through row of dates on top of the card.
3. If at least one of the dates (admission or in treatment) falls within July, separate card onto new pile, otherwise discard the card.

TALLYER:

4. Double check cards separated out with dates for July.

CALLER:

5. Take first card with at least one entry in July.
6. Call out date.
7. Use your finger to trace down the column until you get to the RUTF entries.
8. Call out RUTF amount.

TALLYER:

9. Identify correct week based on date.
10. Note down RUTF amount called out.
11. Repeat amount noted down for caller to check.

Take scans of documents for later verification and entry

- RUTF stock cards for July and weeks/month of visit (November/December)
- Other scans (weekly tallies, monthly reports should have been taken above)

Oxford Policy Management

CIFF-CMAM

Sokoto/Nigeria

Verification of CMAM monitoring data

CMAM OTP data verification collection forms

**LGA NAME**

**VISIT DATE (DD/MM/YY)**

**HEALTH FACILITY NAME**

Circle correct option

OTP Cards available for recount	Y	N
Weekly tally sheets available for capture	Y	N
Monthly site summaries available for capture	Y	N
CMAM register available for recount/capture	Y	N

**COMMENTS:** please comment on any challenges to the verification; discrepancies from the protocol, et.

SECTION 0: COUNTING ADMISSIONS FROM OTP CARD FOR APRIL, MAY, JUNE

	Apr-2017	May-2017	Jun-2017
	**Calendar weeks:**	**Calendar weeks:**	**Calendar weeks:**
Total (Option 1)	TOTAL:	TOTAL:	TOTAL:
By week (Option 2)	Week start/end:	Week start/end:	Week start/end:
	**TOTAL:**	**TOTAL:**	**TOTAL:**
	Week start/end:	Week start/end:	Week start/end:
	**TOTAL:**	**TOTAL:**	**TOTAL:**
	Week start/end:	Week start/end:	Week start/end:
	**TOTAL:**	**TOTAL:**	**TOTAL:**
	Week start/end:	Week start/end:	Week start/end:
	**TOTAL:**	**TOTAL:**	**TOTAL:**
	Week/start end:	Week/start end:	Week/start end:
	**TOTAL:**	**TOTAL:**	**TOTAL:**

SECTION 1-1: TALLYING ADMISSIONS FOR JULY 2017 FROM OTP CARD, 6 - 59 months

	Calendar Week:		Calendar Week:		Calendar Week:		Calendar Week:		Calendar Week:	
	Start/end of week:		Start/end of week:		Start/end of week:		Start/end of week:		Start/end of week:	
Unknown, 6-59 months		TOTAL:		TOTAL:		TOTAL:		TOTAL:		TOTAL:
From self- or community referral, 6-59 months		TOTAL:		TOTAL:		TOTAL:		TOTAL:		TOTAL:
From outpatient care, 6-59 months		TOTAL:		TOTAL:		TOTAL:		TOTAL:		TOTAL:
From inpatient care, 6-59 months		TOTAL:		TOTAL:		TOTAL:		TOTAL:		TOTAL:
ONLY noted relapse, 6-59 months		TOTAL:		TOTAL:		TOTAL:		TOTAL:		TOTAL:
ONLY noted as defaulter, 6-59 months		TOTAL:		TOTAL:		TOTAL:		TOTAL:		TOTAL:
ALSO noted as relapse, 6-59 months		TOTAL:		TOTAL:		TOTAL:		TOTAL:		TOTAL:
ALSO noted as defaulter, 6-59 months		TOTAL:		TOTAL:		TOTAL:		TOTAL:		TOTAL:
INCON-SISTENT DATE ON BACK		TOTAL:		TOTAL:		TOTAL:		TOTAL:		TOTAL:

SECTION 1-2: TALLYING ADMISSIONS FOR JULY 2017 FROM OTP CARD, other ages

	Calendar Week:		Calendar Week:		Calendar Week:		Calendar Week:		Calendar Week:	
	Start/end of week:		Start/end of week:		Start/end of week:		Start/end of week:		Start/end of week:	
Unknown, other ages		**TOTAL:**		**TOTAL:**		**TOTAL:**		**TOTAL:**		**TOTAL:**
From self- or community referral, other ages		**TOTAL:**		**TOTAL:**		**TOTAL:**		**TOTAL:**		**TOTAL:**
From outpatient care, other ages		**TOTAL:**		**TOTAL:**		**TOTAL:**		**TOTAL:**		**TOTAL:**
From inpatient care, other ages		**TOTAL:**		**TOTAL:**		**TOTAL:**		**TOTAL:**		**TOTAL:**
ONLY noted as relapse, other ages		**TOTAL:**		**TOTAL:**		**TOTAL:**		**TOTAL:**		**TOTAL:**
ONLY noted as defaulter, other ages		**TOTAL:**		**TOTAL:**		**TOTAL:**		**TOTAL:**		**TOTAL:**
ALSO noted as relapse, other ages		**TOTAL:**		**TOTAL:**		**TOTAL:**		**TOTAL:**		**TOTAL:**
ALSO noted as defaulter, other ages		**TOTAL:**		**TOTAL:**		**TOTAL:**		**TOTAL:**		**TOTAL:**
INCON-SISTENT DATE ON BACK		**TOTAL:**		**TOTAL:**		**TOTAL:**		**TOTAL:**		**TOTAL:**

SECTION 2: CHECKING SEQUENCE OF TREATMENT DATES AND ABSENCES

Non-consecutive visits		Absence recorded	
	TOTAL:		TOTAL:

TALLYING DISCHARGES

	Calendar week:	Calendar week:	Calendar week:	Calendar week:	Calendar week:
	Start/end of week:	Start/end of week:	Start/end of week:	Start/end of week:	Start/end of week:
Recovered	TOTAL:	TOTAL:	TOTAL:	TOTAL:	TOTAL:
Died	TOTAL:	TOTAL:	TOTAL:	TOTAL:	TOTAL:
Defaulted	TOTAL:	TOTAL:	TOTAL:	TOTAL:	TOTAL:
Not recovered	TOTAL:	TOTAL:	TOTAL:	TOTAL:	TOTAL:
Transferred out	TOTAL:	TOTAL:	TOTAL:	TOTAL:	TOTAL:
Reason unspecified and unclear	TOTAL:	TOTAL:	TOTAL:	TOTAL:	TOTAL:
Unspecified and likely recovered	TOTAL:	TOTAL:	TOTAL:	TOTAL:	TOTAL:
Not discharged but meet criteria for defaulting	TOTAL:	TOTAL:	TOTAL:	TOTAL:	TOTAL:

Calendar week	START-Monday	END-Sunday
Week 13	March 27, 2017	April 2, 2017
Week 14	April 3, 2017	April 9, 2017
Week 15	April 10, 2017	April 16, 2017
Week 16	April 17, 2017	April 23, 2017
Week 17	April 24, 2017	April 30, 2017
Week 18	May 1, 2017	May 7, 2017
Week 19	May 8, 2017	May 14, 2017
Week 20	May 15, 2017	May 21, 2017
Week 21	May 22, 2017	May 28, 2017
Week 22	May 29, 2017	June 4, 2017
Week 23	June 5, 2017	June 11, 2017
Week 24	June 12, 2017	June 18, 2017
Week 25	June 19, 2017	June 25, 2017
Week 26	June 26, 2017	July 2, 2017
Week 27	July 3, 2017	July 9, 2017
Week 28	July 10, 2017	July 16, 2017
Week 29	July 17, 2017	July 23, 2017
Week 30	July 24, 2017	July 30, 2017
Week 31	July 31, 2017	August 6, 2017
Week 32	August 7, 2017	August 13, 2017
Week 33	August 14, 2017	August 20, 2017
Week 34	August 21, 2017	August 27, 2017
Week 35	August 28, 2017	September 3, 2017
Week 36	September 4, 2017	September 10, 2017
Week 37	September 11, 2017	September 17, 2017

Oxford Policy Management

CIFF-CMAM

Sokoto/Nigeria

Verification of CMAM monitoring data

RUTF Verification

**LGA NAME**

**VISIT DATE (DD/MM/YY)**

**HEALTH FACILITY NAME**

Circle correct option

RUTF stock inventory done on day of visit	Y	N
Check of RUTF handed to caregivers done on day of visit	Y	N
Recount of RUTF rations handed out for July	Y	N
RUTF stock card available for capture	Y	N

**COMMENTS:** please comment on any challenges to the verification; discrepancies from the protocol, et.

SECTION 1: CAPTURING RUTF STOCK ON DAY OF VISIT

Calendar week:

	**MORNING COUNT**	
	**Boxes**	**Sachets**
	**EVENING COUNT**	
	**Boxes**	**Sachets**
	**RUTF balance at end of previous week according to RUTF stock card**	
	**Boxes**	**Sachets**
	**RUTF utilised on day of visit according to weekly tally**	
	**Boxes**	**Sachets**
	**RUTF utilised on day of visit according to stock card**	
	**Boxes**	**Sachets**

SECTION 2: RECOUNT RUTF USE ON DAY OF VISIT FROM OTP CARDS

	Sachets disbursed on day
**Total**	

SECTION 3: RUTF received by caregivers, on ration card, on OTP card

Child number	Sachets received	Sachets on ration card	Sachets on OTP card
1			
2			
3			
4			
5			
6			
7			
8			

SECTION 4: TALLYING RUTF FOR JULY 2017 FROM OTP CARDS

	**Calendar Week:**	**Calendar Week:**	**Calendar Week:**
	Start/end of week:	Start/end of week:	Start/end of week:
RUTF rations			
	**Calendar Week:**	**Calendar Week:**	
	Start/end of week:	Start/end of week:	
RUTF rations			

Calendar week	START-Monday	END-Sunday
Week 13	March 27, 2017	April 2, 2017
Week 14	April 3, 2017	April 9, 2017
Week 15	April 10, 2017	April 16, 2017
Week 16	April 17, 2017	April 23, 2017
Week 17	April 24, 2017	April 30, 2017
Week 18	May 1, 2017	May 7, 2017
Week 19	May 8, 2017	May 14, 2017
Week 20	May 15, 2017	May 21, 2017
Week 21	May 22, 2017	May 28, 2017
Week 22	May 29, 2017	June 4, 2017
Week 23	June 5, 2017	June 11, 2017
Week 24	June 12, 2017	June 18, 2017
Week 25	June 19, 2017	June 25, 2017
Week 26	June 26, 2017	July 2, 2017
Week 27	July 3, 2017	July 9, 2017
Week 28	July 10, 2017	July 16, 2017
Week 29	July 17, 2017	July 23, 2017
Week 30	July 24, 2017	July 30, 2017
Week 31	July 31, 2017	August 6, 2017
Week 32	August 7, 2017	August 13, 2017
Week 33	August 14, 2017	August 20, 2017
Week 34	August 21, 2017	August 27, 2017
Week 35	August 28, 2017	September 3, 2017
Week 36	September 4, 2017	September 10, 2017
Week 37	September 11, 2017	September 17, 2017
